# Pan-cancer multi-omics analysis reveals upregulation of the POLE2 subunit of DNA polymerase epsilon and its associations with patient survival and cellular pathways beyond DNA replication

**DOI:** 10.1093/nargab/lqag118

**Published:** 2026-10-14

**Authors:** Alina Krasilia, Deepali Chaudhry, Iwona J Fijalkowska, Michal Dmowski

**Affiliations:** Institute of Biochemistry and Biophysics, Polish Academy of Sciences, Pawinskiego 5a, 02-106 Warsaw, Poland; Institute of Biochemistry and Biophysics, Polish Academy of Sciences, Pawinskiego 5a, 02-106 Warsaw, Poland; Doctoral School of Molecular Biology and Biological Chemistry, Warsaw, Poland; Institute of Biochemistry and Biophysics, Polish Academy of Sciences, Pawinskiego 5a, 02-106 Warsaw, Poland; Institute of Biochemistry and Biophysics, Polish Academy of Sciences, Pawinskiego 5a, 02-106 Warsaw, Poland

## Abstract

DNA polymerase epsilon (Polε) is essential for high-fidelity leading-strand synthesis. It comprises a catalytic subunit, POLE, and three accessory subunits, including POLE2. In contrast to POLE, the biological functions and clinical significance of POLE2 remain unexplored. POLE2 is vital for the assembly of Polε and for interactions with the CMG helicase. Yeast studies show that dysfunction of its ortholog, Dpb2, causes genomic instability and impaired cell cycle progression. To investigate the significance of POLE2, this study provides a comprehensive multi-omics analysis across tumor types. We show that POLE2 upregulation is associated with decreased overall survival in a cancer-type-specific manner. Its expression levels, rather than copy number variation (CNV), emerge as a more reliable prognostic marker. POLE2 expression regulatory mechanisms include promoter methylation, isoform usage, and epigenetic regulators. Elevated POLE2 messenger RNA levels rarely translate into increased protein levels, possibly because of abundant non-coding isoforms. Co-expression and pathway enrichment analyses reveal an association between POLE2 and transcriptional regulation involving E2F, EZH2, and RB. Our findings highlight POLE2 as a contributor to genome stability, a candidate cancer biomarker, and a possible therapeutic target.

## Graphical Abstract

**Graphical Abstract:**
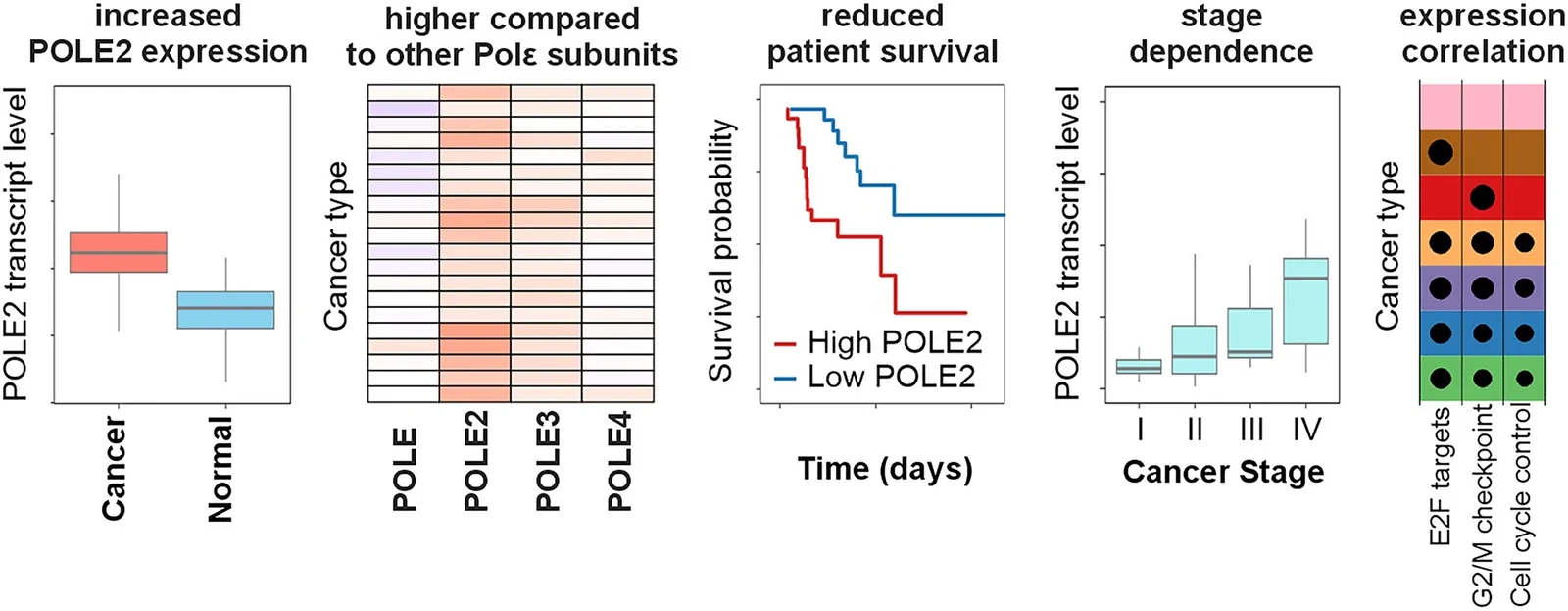

## Introduction

Faithful DNA replication is essential for preserving genomic integrity, and its disruption is a major contributor to mutagenesis and oncogenesis. DNA polymerase epsilon (Polε) is one of the key enzymes involved in high-fidelity DNA synthesis, primarily responsible for leading-strand replication [1]. It comprises a catalytic subunit (POLE) and three accessory subunits—POLE2, POLE3, and POLE4—involved in the holoenzyme complex assembly and function [2–5]. While the catalytic subunit POLE has been extensively studied, particularly in its proofreading exonuclease domain and its role in hypermutated tumors, the functions of non-catalytic subunits of Polε remain less understood.

Although it lacks enzymatic activity, POLE2 is required for viability in yeast and mammalian cells [6–8]. Moreover, POLE2 is essential for the assembly and stability of the Polε holoenzyme, i.e., for anchoring the catalytic core to accessory subunits [6–8] and for stabilizing interactions with the replicative helicase, the CMG (Cdc45-MCM-GINS) complex [2, 5, 9]. Structural studies have shown that POLE2 is highly conserved from yeast (Dpb2) to humans, indicating a fundamental role in eukaryotic DNA replication [6, 7, 10, 11]. Dysregulation or mutation of POLE2 may impair the cooperation between Polε and the CMG helicase complex, potentially leading to replication stress, increased mutation rates, and genomic instability—hallmarks of cancer.

So far, our knowledge of the effects of dysfunction of the main accessory subunit of Polε comes from studies of the yeast ortholog of POLE2, *DPB2*. In our previous studies, it has been shown that mutations in *DPB2*, which affect the interactions between the polymerase and helicase complexes, result in strong genomic instability. This involves increased mutation rates, impaired progression through the S phase, abnormal cell morphology, defective replication checkpoint activation, and increased instability of DNA repeat tracts [12–17]. The impaired functioning of the replisome in Polε-defective cells is at the source of perturbations in helicase-polymerase function, severely affecting DNA replication and resulting in the formation of single-stranded DNA. This necessitates a rescue by DNA repair mechanisms, mainly those related to homologous recombination [18]. Moreover, it affects the division of labor between the two main DNA polymerases, resulting in an increased contribution of Pol δ to leading strand replication [19].

Recent reports have identified a range of POLE2 alterations in various tumor types, including colorectal, endometrial, renal, biliary, and breast cancers [20–26]. These alterations include missense and nonsense mutations, frameshift insertions and deletions, silent mutations with potential regulatory impact, and large-scale structural variations. Moreover, changes in POLE2 mRNA expression and epigenetic modifications, such as promoter methylation, have also been documented; however, their functional implications are not yet fully understood. 

This study presents a comprehensive analysis of POLE2 alterations and their potential clinical and functional consequences. We evaluate the association between POLE2 status and patient overall survival (OS) to determine whether POLE2 alterations may serve as prognostic markers. Additionally, we assess POLE2 gene expression, DNA methylation status, and alternative isoform usage, exploring how these layers of regulation may influence cancer phenotype. We also analyze the spectrum of POLE2 mutations across multiple cancers using publicly available datasets. To understand the broader impact of POLE2 dysregulation, we perform genetic alteration analysis, identify co-expressed genes, and carry out enrichment analysis of associated pathways and biological processes. Through this integrative multi-omics approach, we aim to elucidate the role of POLE2 in cancer biology, its potential contribution to genome instability, and its relevance as a biomarker or therapeutic target. Given the central importance of DNA replication in oncogenesis, a deeper understanding of POLE2 may uncover novel mechanisms of tumor development and progression.

## Materials and methods

### Overall survival analysis based on POLE2 molecular alterations

To assess the association between POLE2 expression and overall survival (OS), four independent platforms based on The Cancer Genome Atlas (TCGA) were employed: University of California, Santa Cruz (UCSC Xena) (https://xena.ucsc.edu/) [27], Encyclopedia of RNA Interactomes (ENCORI) (https://rnasysu.com/encori/) [28], GEPIA (Gene Expression Profiling Interactive Analysis) (http://gepia2.cancer-pku.cn/) [29], and Tumor-Immune System Interactions Database (TISIDB) (http://cis.hku.hk/TISIDB/) [30]. Among these, UCSC Xena was used for pan-cancer analysis and further individual cancer-type-specific analysis. In the initial pan-cancer analysis, the Kaplan–Meier (KM) Plot option in UCSC Xena was used to evaluate survival associations using POLE2 CNV, average mRNA expression across all cancer types, and mutation status. This database stratifies groups based on the median expression value. Following this, a second stage of analysis was performed at the level of individual cancer types. Initially, the KM Plot option in UCSC Xena was used for each TCGA cancer type (listed in Table 1) to evaluate the significance of the association between POLE2 expression and OS. To validate and reinforce the findings, results were compared against three additional platforms: ENCORI  via its Pan-Cancer Survival Analysis module to identify cancer types showing statistically significant associations (*P* < .05) between POLE2 expression and OS; GEPIA2 using OS analysis with a 50% expression cutoff to define high- and low-expression cohorts; and TISIDB through its Clinical module for OS assessment across TCGA datasets in relation to POLE2 expression.

**Table 1.** Abbreviations and full names of cancer types from TCGA

Abbreviation	Cancer type
ACC	**A**dreno**c**ortical **c**arcinoma
BLCA	**Bl**adder Urothelial **Ca**rcinoma
BRCA	**Br**east invasive **ca**rcinoma
CESC	**Ce**rvical **s**quamous cell **c**arcinoma and endocervical adenocarcinoma
CHOL	**Chol**angiocarcinoma
COAD	**Co**lon **ad**enocarcinoma
DLBC	Lymphoid Neoplasm **D**iffuse **L**arge **B**-**c**ell Lymphoma
ESCA	**Es**ophageal **ca**rcinoma
GBM	**G**lio**b**lastoma **m**ultiforme
HNSC	**H**ead and **N**eck **s**quamous cell **c**arcinoma
KICH	**Ki**dney **Ch**romophobe
KIRC	**Ki**dney **r**enal clear cell **c**arcinoma
KIRP	**Ki**dney **r**enal **p**apillary cell carcinoma
LAML	**A**cute **M**yeloid **L**eukemia
LGG	Brain **L**ower **G**rade **G**lioma
LIHC	**Li**ver **h**epatocellular **c**arcinoma
LUAD	**Lu**ng **ad**enocarcinoma
LUSC	**Lu**ng **s**quamous cell **c**arcinoma
MESO	**Meso**thelioma
OV	**Ov**arian serous cystadenocarcinoma
PAAD	**Pa**ncreatic **ad**enocarcinoma
PCPG	**P**heo**c**hromocytoma and **P**ara**g**anglioma
PRAD	**Pr**ostate **ad**enocarcinoma
READ	**Re**ctum **ad**enocarcinoma
SARC	**Sarc**oma
SKCM	**Sk**in **C**utaneous **M**elanoma
STAD	**St**omach **ad**enocarcinoma
TGCT	**T**esticular **G**erm **C**ell **T**umors
THCA	**Th**yroid **ca**rcinoma
THYM	**Thym**oma
UCEC	**U**terine **C**orpus **E**ndometrial **C**arcinoma
UCS	**U**terine **C**arcino**s**arcoma
UVM	**Uv**eal **M**elanoma

TCGA cancer types that showed consistent and significant OS associations with POLE2 expression across all four platforms were selected for focused cancer-specific survival analysis, both uni- and multivariable. For this, the UCSC Xena database was used to export cancer-specific data and OS data, which were analyzed in RStudio (R Core Team (2024) R: A Language and Environment for Statistical Computing R Foundation for Statistical Computing, Vienna, Austria; https://www.r-project.org/), employing the *survival* package (10.32614/CRAN.package.survival) [31]. The Surv() function was used to define survival time and event status. KM curves were fitted using survfit(), and group differences were evaluated with the log-rank test via survdiff(). *P*-values were calculated from the resulting chi-squared statistics using pchisq(). To quantify the effect of POLE2 expression on OS, a Cox proportional hazards regression model was fitted using coxph(). POLE2 expression group (“High” versus “Low”) was used as a categorical explanatory variable. The model estimated the hazard ratio (HR) and its 95% confidence interval (CI).

To account for cancer-specific survival factors in the multivariable Cox proportional hazards regression models, clinical and histopathological covariates were tailored to the standard prognostic metrics of each respective cohort. For adrenocortical carcinoma (ACC), the model was adjusted for pathologic stage and Weiss score, which serves as a standard system for evaluating the malignant potential of adrenal tumors. In the brain lower-grade glioma (LGG) cohort, because primary brain tumors do not utilize traditional TNM staging, the model was instead adjusted for histologic grade and IDH1 mutation status; notably, to overcome incomplete default database annotations, true IDH1 mutation status was manually derived from raw genomic mutation data to maximize sample inclusion. For lung adenocarcinoma (LUAD), the model was adjusted for AJCC pathologic stage, which is the primary clinical determinant of baseline survival in lung cancers. Finally, the mesothelioma (MESO) model was adjusted for both pathologic stage and histological type, as survival outcomes in this disease are heavily dependent on specific cellular morphology—such as epithelioid or sarcomatoid classifications—in addition to anatomical spread. Visualization was performed using the *ggplot2* package [32], and multiple plots were arranged using wrap_plots() from *patchwork* (10.32614/CRAN.package.patchwork) for figure composition. For most of the graphs, theme_few() from the package *ggthemes* (https://github.com/jrnold/ggthemes) was used.

To evaluate the prognostic impact of specific transcript variants, *POLE2* isoform data were integrated into the survival analysis pipeline. As defined in “Isoform expression and transcript analysis,” individual transcripts were classified based on their annotated biological functions into three distinct groups: coding, non-coding, and problematic. For each patient sample, the relative expression percentages of the transcripts comprising each functional group were summed. These aggregated percentages were then multiplied by the sample’s total *POLE2* gene expression to derive a specific expression estimate for each respective category (coding expression, non-coding expression, and problematic expression). Consistent with the primary OS analysis, patients were stratified into “High” and “Low” cohorts for each of the three isoform groups using the median expression value as the cutpoint. The prognostic significance of each isoform group was independently evaluated using univariable Cox proportional hazards regression. To determine if the survival associations of these specific isoform categories were independent of standard clinical factors, they were subsequently evaluated using multivariable Cox regression models, adjusting for the identical cancer-specific covariates (e.g., pathologic stage, histologic grade, and IDH1 mutation status) established previously.

### mRNA expression analysis

Average mRNA and exon expression of POLE2 in cancer and normal TCGA PANCAN datasets were downloaded from UCSC Xena (https://xena.ucsc.edu/). Alongside general expression analysis, POLE2 mRNA levels in different CNV groups were analyzed, particularly from the “gistic2_thresholded” dataset, which includes only tumor samples, while only the “Primary tumor” sample type was taken into analysis. In this dataset, CNV groups were divided into five categories: −2 represents a homozygous deletion, where both gene copies are likely lost, although some mRNA expression may persist due to normal cell contamination or technical artifacts. −1 indicates a heterozygous deletion, with one copy lost. 0 corresponds to a normal (diploid) copy number. +1 denotes a low-level gain, typically one extra copy, while +2 indicates a high-level amplification involving multiple additional copies. These simplified categories approximate underlying genomic alterations but may not fully capture accurate gene dosage.

The significance of the difference between groups was calculated using RStudio. Pairwise comparisons between cancer and normal sample types were conducted using the Wilcoxon rank-sum test. The Benjamini–Hochberg correction was applied to control for false discovery due to multiple comparisons. The analysis was performed using the pairwise.wilcox.test() function from the *rstatix* (https://kassambara.r-universe.dev/rstatix) package. The stat_pvalue_manual() function from the *ggpubr* [38] package was used to visualize significance on the plot.

For cancer-specific expression analysis, the UCSC Xena database was used as a source of expression data, while other databases were used to validate cancer types that consistently show significant differences between cancer and normal samples. GEPIA2 (http://gepia2.cancer-pku.cn/) [29] database, with and without including GTEx normal tissue data, was used via the “Expression DIY → Box Plot” panel. A *P*-value cutoff of .05 and a log_2_ fold change (log_2_FC) of 1 were used. GEPIA2 applies a one-way analysis of variance model, treating the disease state (tumor versus normal) as the grouping variable. Expression values were log-transformed as log_2_(TPM + 1) prior to analysis. Cancer types that resulted in significant changes between cancer and normal were noted. Additionally, ENCORI (https://rnasysu.com/encori/) [28] Pan-Cancer Differential Expression Analysis option was used to assess TCGA cancer types with a significant difference in POLE2 expression between cancer and normal samples. Similarly, UALCAN (University of Alabama at Birmingham Cancer Data Analysis Portal; https://ualcan.path.uab.edu/analysis.html) [33, 34] was used for the same purpose. To further validate the data, the TIMER2.0 (http://timer.comp-genomics.org/) [35, 36] database “Cancer Exploration→ Gene_DE” panel was used, and cancer types with *P *< .05 were selected.

TCGA data from the UCSC Xena database were used to visualize selected cancer types. Statistical comparisons between cancer and normal samples were performed using the wilcox.test() function from the *rstatix* package. Visualization was performed as described above.

For visualization of POLE2 expression in different tissues, the data were collected using the getTissueVST() function from the *correlationAnalyzeR* package [37]. Statistical comparisons between cancer and normal samples were performed using the wilcox.test() function from the *rstatix* package. Effect sizes were assessed using two metrics: log_2_FC to quantify the magnitude of expression change, and Cohen’s d to measure the standardized mean difference between cancer and normal samples. Cohen’s d was computed using the cohen.d() function from the effsize package (10.32614/CRAN.package.effsize) with a 95% CI. Cohen’s d represents the standardized mean difference between two groups, expressed in units of pooled standard deviation. Visualization was performed as described above.

Gene expression data from TCGA and GTEx were obtained from the UCSC Xena database to compare the expression of genes involved in DNA replication and repair pathways across various TCGA cancer types. GTEx samples were matched to corresponding TCGA cancer types using a manually curated tissue concordance table, based on the “primary_site” metadata provided by UCSC Xena. Differential expression analysis was performed using the limma [45] package for each cancer type. Expression matrices were transposed so that genes were rows and samples were columns. A contrast model comparing cancer to normal samples was fit using the lmFit() and eBayes() functions. All genes were tested, and the log_2_FC, raw p-value, and adjusted p-value (using the Benjamini–Hochberg method) were calculated for each. To visualize differential expression patterns of genes involved in DNA replication and repair, a heatmap was generated using *ggplot2* [32]. Genes were grouped into functional categories (e.g., MCM, GINS, Pol ε, CDC45), and each group was assigned a consistent color. Gene names were displayed in color using markdown formatting via *ggtext* (https://wilkelab.org/ggtext/), and a custom legend indicating gene group membership was added alongside the plot using the *cowplot* (10.32614/CRAN.package.cowplot) package.

POLE2 mRNA expression across tumor stages was analyzed using batch effect–normalized RNA-seq expression (RSEM) data from the UCSC Xena database and cBioPortal (https://www.cbioportal.org/) [38–40]. In the UCSC Xena TCGA dataset, "ajcc_pathologic_tumor_stage" was used as the phenotypic stage annotation alongside POLE2 expression data, which were converted from log_2_(norm + 1) format back to normalized RSEM counts. Tumor stage codes were standardized to a numeric scale from 1 to 4. In cBioPortal, the TCGA PanCancer Atlas Studies were selected as the data source. POLE2 expression values, already provided on a linear scale, were analyzed against the clinical attributes of the American Joint Committee on Cancer Tumor Stage Code. Starting from this point, both datasets were processed identically. Outliers were removed within each cancer type and stage using the interquartile range method. Cancer types with significant stage-specific differences in POLE2 expression were identified using the Kruskal–Wallis test, implemented via the kruskal_test() function from the rstatix package. For these cancer types, pairwise Wilcoxon rank-sum tests (pairwise_wilcox_test()) were performed to compare expression levels between stages, with *P*-values adjusted using the Benjamini–Hochberg method.

For protein and mRNA expression comparison, the UALCAN portal was used to export CPTAC plot data for cancer types comparable with TCGA cancers, together with TCGA plot data for the same cancer types. Visualization was performed as described above.

### POLE2 methylation analysis

DNA methylation for the POLE2 promoter region from the Illumina HumanMethylation450 BeadChip (450K array) dataset was downloaded from the UCSC Xena (https://xena.ucsc.edu/) [27] database. Gene average methylation and detailed view data were exported together with cancer type abbreviations and sample type data. Methylation levels (β-values) between cancer and normal samples were compared using the Wilcoxon rank-sum test, implemented via the wilcox.test() function from the *rstatix* package. For comparison between CpG sites and cancer types, *P*-values were adjusted using the Benjamini–Hochberg method. The mean methylation difference (Δβ) was calculated as the difference in average β-values between cancer and normal groups. Visualization was performed as described above.

The location of reported CpG sites relative to the POLE2 sequence was exported from the UCSC Genome Browser (https://genome.ucsc.edu/) [41]. The human (GRCh37/hg19) genome assembly was used for analysis. CpG site coordinates were obtained from the “Illumina 450K Methylation Array” subtrack under the “Array Probesets” track [42]. CpG island locations were retrieved from the “CpG Islands” track [43]. Gene annotation for POLE2 was based on the “GENCODE V47lift37” track [44], and functional domain information, including the N-terminal domain of POLE2, was obtained from the “Pfam in UCSC Gene” track [45].

### Isoform expression and transcript analysis

TOIL RSEM isoform percentage data from the UCSC Xena database were used for isoform expression analysis. Isoform percentage and sample type data were exported from the TCGA and GTEx datasets. GTEx normal tissue data were assigned to corresponding TCGA cancer type abbreviations for comparison, as detailed in Supplementary Table S1. For the Cancer category, only samples labeled as “Primary Tumor” from TCGA were used. The Normal_TCGA category included “Solid Tissue Normal” samples, representing tissue adjacent to tumors. The Normal_GTEx category included “Normal” samples from GTEx, representing normal tissues unrelated to tumors. Isoform structure data were exported from the UCSC Genome Browser, using the Comprehensive Gene Annotation Set from GENCODE Version 29 (Ensembl 94) based on the human genome assembly GRCh38/hg38. Transcripts were classified based on their annotated biological function as coding (protein-coding and polymorphic pseudogenes), non-coding (non-protein-coding transcripts), or problematic (biotypes including retained_intron, TEC, or disrupted_domain) [46]. Isoform expression levels for each TCGA cancer type were exported from GEPIA 2.0.

### Genetic alteration analysis

A systematic analysis of POLE2 mutations across cancer types was conducted using data from the COSMIC (https://www.cosmickb.org/) and the UCSC Genome Browser (human GRCh38/hg38) (https://genome.ucsc.edu/) [41]. The POLE2 gene was first aligned with the COSMIC mutation track (Version 101) [47] in the UCSC Genome Browser to extract mutation coordinates. Descriptive data were then obtained from the COSMIC database for the ENST00000216367.9 transcript (POLE2-001), including information on point mutation positions, amino acid changes, and associated tissue types (Supplementary Table S2). Exon-intron coordinates were obtained from the UCSC Genome Browser for the same transcript. Somatic mutations with a recurrence count greater than one were visualized using *ggplot2* [32] and *cowplot* packages in R (10.32614/CRAN.package.cowplot).

### Correlation analysis

Lists of genes with high POLE2 Pearson correlation value (>0.8) were exported from four independent databases, where Correlation AnalyzeR (https://gccri.bishop-lab.uthscsa.edu/shiny/correlation-analyzer/) [37] contained data both for cancer and normal samples, UALCAN (University of Alabama at Birmingham Cancer Data Analysis Portal, https://ualcan.path.uab.edu/analysis.html) [33, 34] and GEPIA2 (http://gepia2.cancer-pku.cn/) [29] provided data only for cancer patients; and GTExPortal (https://gtexportal.org/home/) provided data exclusively for normal tissue. Initial data sources were TCGA data for UALCAN and GEPIA2, ARCHS4 for Analyzer, and GTEx original data for GTExPortal. In the Correlation AnalyzeR database, POLE2-correlated genes were exported for available tissue types with both cancer and normal sample data. The UALCAN database provides POLE2-correlated genes for TCGA cancer samples through the “Correlation” section. The GEPIA2 database offers the top 1000 genes most similar to POLE2 in TCGA tumor samples, available under the “Expression Analysis → Similar Genes Detection” tab. To get POLE2 correlation values for normal tissues, open-access data from GTExPortal were downloaded, namely GTEx Analysis V10 RNA-seq data (GTEx_Analysis_2022–06-06_v10_RNASeQCv2.4.2_gene_tpm_non_lcm.gct) with metadata (GTEx_Analysis_v10_Annotations_SampleAttributesDS.txt). Sample IDs were extracted for each tissue, and gene expression data were filtered to include only relevant samples (Supplementary Table S3).

Correlation between POLE2 expression and all other genes was computed using Pearson correlation for each tissue using the cor.test() function. *P*-values for the correlations were calculated, and genes with *P* < .05 and |correlation| > 0.8 were retained. Tissues were grouped into functional categories based on anatomical and biological relevance using a predefined mapping table (Supplementary Table S4).

Gene biotypes were determined from the GENCODE v47 Basic Annotation GTF file. The GTF file was parsed to extract gene_name and gene_type, retaining unique entries. Genes annotated as “protein-coding” were selected for downstream analysis.

To visualize the overlap between POLE2-correlated or similar genes obtained from multiple datasets (UALCAN, GEPIA2, GTEx, and Analyzer), the UpSet plot was generated using the upset() function from the ComplexUpset package (10.5281/zenodo.3700590) [48], with “exclusive_intersection” mode. Intersections containing at least one gene were extracted using the upset_data() function from the ComplexUpset package and were used for subsequent enrichment analysis.

### Enrichment analysis

Over-representation analysis (ORA) was performed to identify pathways enriched among genes uniquely shared across dataset intersections. Genes from each exclusive intersection were tested against predefined gene sets (e.g., Hallmark pathways) using the enricher() function from the clusterProfiler package [49] with a false discovery rate threshold (*q*-value cutoff) of 0.2. *P*-values were adjusted using the Benjamini–Hochberg method to control for multiple testing. Gene set annotations were loaded using read.gmt() from clusterProfiler. Significant results (adjusted *P*-value < .05) were visualized as dot plots with ggplot2 as described above. Predefined gene sets in .gmt format were downloaded from MSigDB (Molecular Signatures Database) (https://www.gsea-msigdb.org/gsea/msigdb) [50], specifically from “Human MSigDB v2024.1.Hs” updated August 2024. In Fig.10, the “h.all.v2024.1.Hs.symbols.gmt” [51] file was used, whereas in Fig. 11B, the “h.all.v2024.1.Hs.symbols.gmt,” “c2.cp.kegg_legacy.v2024.1.Hs.symbols.gmt,” “c6.all.v2024.1.Hs.symbols.gmt,” and “c3.tft.tft_legacy.v2024.1.Hs.symbols.gmt” [51, 52] files were used. Enriched terms and their genes were visualized with the *plotly* package [53] and saved with the *webshot2* package (10.32614/CRAN.package.webshot2).

Functional enrichment analysis was performed using the Metascape (http://metascape.org) [54] resource based on exclusively cancer-related gene sets for separate TCGA cancer types. Circos plot visualization and protein–protein interaction network (PPI network, Physical Core option) were generated by Metascape, using the Molecular Complex Detection (MCODE) algorithm, based on data from STRING, BioGrid, OmniPath, and InWeb_IM. PPI network was edited in Cytoscape [55].

## Results and discussion

### Survival analysis reveals the prognostic significance of POLE2 expression in cancer

To assess how POLE2 perturbations affect overall survival (OS) of cancer patients, KM plots were generated for POLE2 CNV, mRNA expression, and mutations (Fig. 1A). The results show that OS was significantly lower in patients with higher POLE2 mRNA expression (high expression group: *n* = 4828; low expression group: *n* = 4814; *P*-value < 2.2e-16). In contrast, no significant differences in survival rates were observed between groups stratified by POLE2 CNV (high CNV group: *n* = 4931; low CNV group: *n* = 4883; *P*-value = .5418) or mutation status (mutation group: *n* = 59; no mutation group: *n* = 8627; *P*-value = .6421). Notably, POLE2 mutations were rare across the cohort, and the small number of mutated cases compared with the non-mutated group makes it difficult to draw definitive conclusions about the impact of mutations on survival.

**Figure 1.**
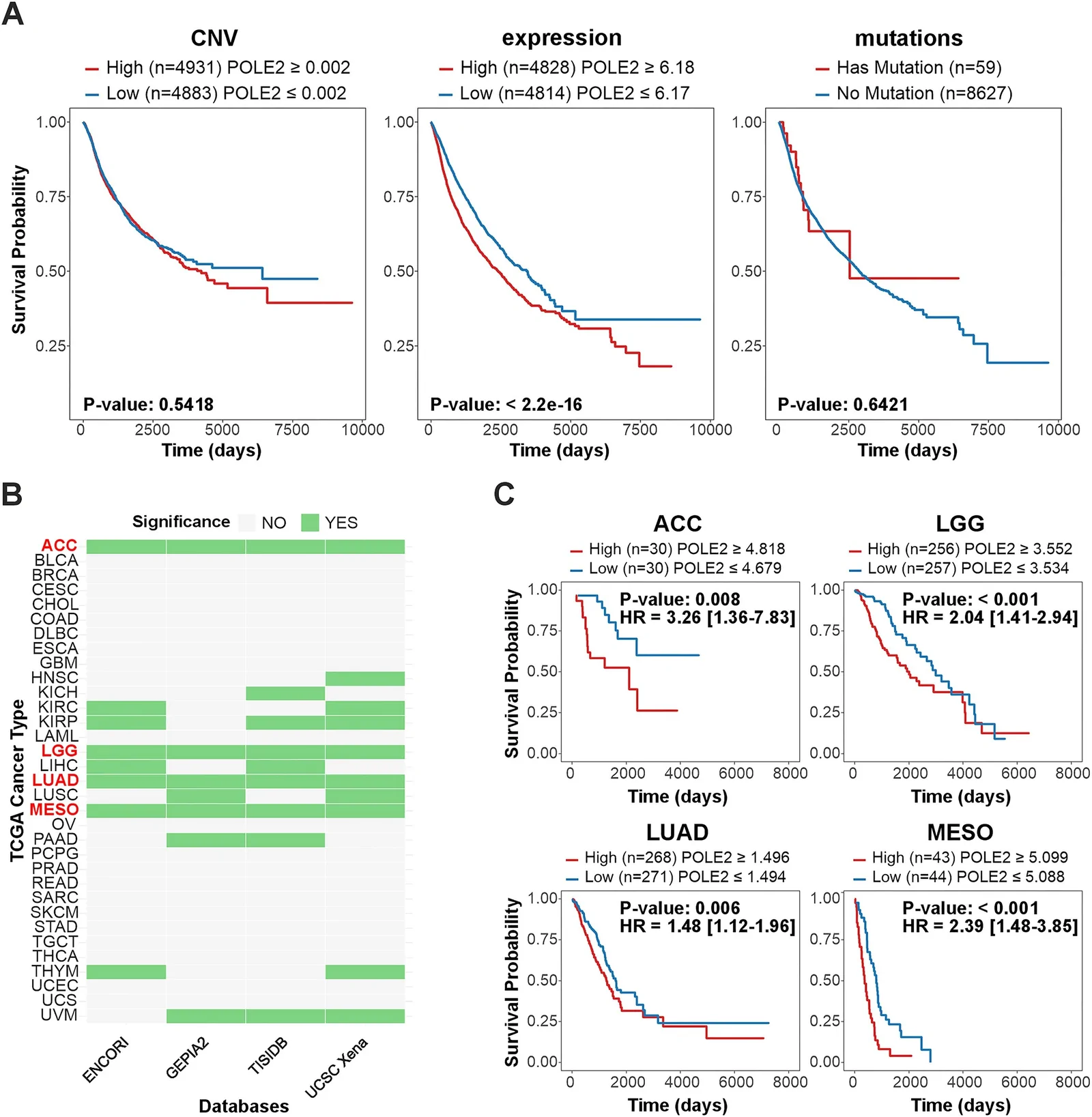
OS analysis of patients based on POLE2 alterations. (**A**) KM survival plots from TCGA Pan-Cancer (PANCAN) data from the UCSC Xena database assessing the impact of POLE2 CNV, mRNA expression levels, and mutation status on patient OS. (**B**) Heatmap summarizing the statistical significance (*P* ≤ .05) of POLE2 mRNA expression-associated survival across multiple TCGA cancer types, evaluated using independent databases (ENCORI, TISIDB, GEPIA2, UCSC Xena). Cancer types consistently showing significant associations across all platforms are highlighted in red. (**C**) KM plots from TCGA Pan-Cancer (PANCAN) data from the UCSC Xena database for selected cancer types from panel B (ACC, LGG, LUAD, and MESO), in which high POLE2 mRNA levels consistently correlate with OS outcomes across all datasets. The plot displays the HR followed by its 95% CI from Cox proportional hazards models in square brackets. For example, “HR: 3.54 [1.58–7.92]” indicates that patients with high POLE2 expression had a 3.54 times higher risk of death compared to those with low expression, with the true value likely falling between 1.58 and 7.92.

These findings suggest that POLE2 expression level, rather than CNV or mutation status, may serve as a potential prognostic marker for cancer patients. However, CNV and mutation may still exert indirect effects by influencing gene expression or through non-survival-related mechanisms. These aspects have been further explored in subsequent sections of the analysis, where we examine the relationship between CNV and POLE2 expression, and the frequency and potential functional impact of POLE2 mutations across cancer types.

To identify particular cancer types in which survival is significantly associated with POLE2 mRNA level, data from 33 TCGA cancer types (listed in Table 1) were collected from four independent databases (UCSC Xena, ENCORI, GEPIA, and TISIDB) and compared (Fig. 1B). Patients were divided into high and low POLE2 expression groups based on cancer-type-specific thresholds that equal the median POLE2 expression value. The analysis revealed four cancer types—ACC, LGG, LUAD, and MESO—consistently showing a significant (*P* ≤ .05) survival difference between groups stratified by POLE2 mRNA level. Separate KM plots for these cancer types are presented in Fig. 1C. The HR indicates the relative risk of death in the high-expression group compared to the low-expression group. The strongest effect was observed in ACC, where high POLE2 expression was associated with significantly poorer survival (HR: 3.26 [1.36–7.83], *P* = .008). MESO also showed a strong association (HR: 2.39 [1.48–3.85], *P* = .0002), followed by LGG (HR: 2.04 [1.41–2.94], *P* = .0002). A weaker but still significant association was found in LUAD (HR: 1.48 [1.12–1.96], *P* = .006). Several other cancer types also demonstrated partial evidence linking POLE2 mRNA levels to survival outcomes. Specifically, KIRP and UVM showed supportive results in 3 out of 4 databases; KIRC, LIHC, LUSC, PAAD, and THYM showed positive associations in 2 out of 4 databases; HNSC and KICH showed associations in 1 out of 4 databases. Separate KM plots for these cancers can be found in Supplementary Fig. S1. Notably, KICH and UVM exhibited strong HRs (HR: 3.44 [0.71–16.56], HR: 2.95 [1.16–7.51], respectively), although only UVM reached statistical significance, while the wide CI in KICH reflects uncertainty due to a small sample size. A similarly elevated HR was seen in KIRP (1.96 [1.05–3.66], where the association was statistically significant, indicating a nearly twofold increase in risk. Moderate but significant HRs around 1.4 were found in KIRC and LIHC, indicating a weaker yet consistent increase in risk with higher POLE2 expression. In contrast, THYM demonstrated an HR well below 1 (0.18 [0.30–0.89], suggesting a protective effect of high POLE2 expression, which was also statistically significant. LUSC showed a similar inverse trend (HR: 0.75 [0.57–0.99], though the effect size was smaller. For PAAD and HNSC, HRs close to 1 (1.35 and 0.81, respectively) indicated little to no association, and these findings were not statistically significant.

These findings demonstrate that elevated POLE2 expression is associated with increased HRs and decreased OS, with the strength and direction of this association being cancer-type specific. The most pronounced and consistently significant effects were observed in ACC, LGG, LUAD, and MESO. While the univariate findings established high *POLE2* expression as a consistent predictor of poor OS, multivariable analysis integrating cancer-specific clinical parameters chosen for each cohort, as outlined in the methods section, reveals a stark divergence in its independent prognostic value (Table 2). When adjusting for tailored covariates such as Weiss criteria in ACC, tumor stage in LUAD, and histological type in MESO, the elevated mortality risk associated with *POLE2* remained statistically significant and structurally stable (ACC: HR = 3.33, *P* = .019; LUAD: HR = 1.41, *P* = .017; MESO: HR = 2.35, *P* = .001). In contrast, *POLE2* entirely lost prognostic significance in the LGG cohort (HR = 1.22, non-significant) once the model accounted for tumor grade and IDH mutation status.

**Table 2.** Univariate and multivariate Cox regression analyses of POLE2 expression and clinicopathological variables for OS in ACC, LGG, LUAD, and MESO cohorts

Characteristic	Cohort distribution	Univariate analysis			Multivariate analysis		
		HR	95% CI	*P*-value	HR	95% CI	*P*-value
**ACC**	***N* = 60**						
POLE2_expression high/low	30 / 30	3.26	1.36-7.83	**0.008**	3.33	1.22-9.07	**0.019**
Stage II / I	8 / 27	2.22	0.27-18.5	0.5	2.04	0.24-17.1	0.5
Stage III / I	8 / 11	7.40	0.85-64.2	0.07	3.91	0.43-35.9	0.2
Stage IV / I	8 / 14	18.2	2.22-150	**0.007**	17.5	2.07-147	**0.009**
Weiss group high/low	30 / 30	2.03	0.87-4.77	0.1	0.80	0.31-2.06	0.6
**LGG**	***N* = 513**						
POLE2 expression high/low	257 / 256	2.04	1.41-2.94	**<0.001**	1.22	0.81-1.82	0.3
Grade G3 / G2	248 / 265	3.43	2.31-5.10	**<0.001**	2.58	1.71-3.89	**<0.001**
IDH status Yes / No	113/395	0.23	0.16-0.33	**<0.001**	0.31	0.21-0.47	**<0.001**
**LUAD**	***N* = 539**						
POLE2 expression high/low	271 / 268	1.48	1.12-1.96	**0.006**	1.41	1.06-1.88	**0.017**
Stage II / I	298 / 122	2.19	1.53-3.13	**<0.001**	2.13	1.49-3.05	**<0.001**
Stage III / I	298 / 92	3.27	2.29-4.66	**<0.001**	3.22	2.26-4.60	**<0.001**
Stage IV / I	298 / 27	3.46	2.06-5.81	**<0.001**	3.43	2.04-5.75	**<0.001**
**MESO**	***N* = 87**						
POLE2 expression high/low	44 / 43	2.39	1.48-3.85	**<0.001**	2.35	1.41-3.93	**0.001**
Stage II / I	10 / 16	0.63	0.26-1.51	0.3	0.92	0.37-2.34	0.9
Stage III / I	10 / 45	0.76	0.36-1.59	0.5	0.78	0.36-1.73	0.5
Stage IV / I	10 / 16	0.71	0.30-1.67	0.4	0.71	0.29-1.75	0.5
Biphasic / Epithelioid mesothelioma	57 / 23	2.01	1.19-3.41	**0.009**	1.90	1.10-3.27	**0.021**
Sarcomatoid / Epithelioid mesothelioma	57 / 2	6.87	0.89-52.9	0.064	3.88	0.45-33.4	0.2
Diffuse malignant / Epithelioid mesothelioma - NOS	57 / 5	1.16	0.46-2.93	0.8	1.13	0.44-2.90	0.8

Abbreviations: CI = confidence interval, HR = hazard ratio.

Further examination of KM curves and HRs in cancer types showing significance in at least one external database indicated that UVM may also have prognostic relevance. Additionally, KIRC and LIHC displayed HRs comparable to those of LUAD, suggesting similar risk patterns linked to high POLE2 expression. This observation aligns with previous findings in renal cell carcinoma (RCC), of which KIRC is the most common subtype, where high POLE2 expression has been associated with reduced overall and progression-free survival [21]. Notably, although high POLE2 expression has previously been reported to correlate with poor survival in bladder cancer (BLCA) [26], our data did not support a significant association in this cancer type. In contrast, THYM and LUSC showed an inverse relationship, where elevated POLE2 expression was associated with improved survival, indicating a potential protective effect.

The strategy of cross-validating results across multiple independent databases proved effective, identifying three of the four cancer types with the strongest effects and reinforcing the observed associations’ reliability.

### POLE2 expression analysis in cancer and normal samples

To explore how different alterations in POLE2 gene expression differ between cancer and normal tissues, we first analyzed POLE2 mRNA expression levels and exon-level expression profiles using data aggregated across all TCGA cancer types. The statistical comparison of mRNA levels and exon expression of the POLE2 gene revealed that there is a significant difference between cancer and normal samples, with an increase in cancer cells (Fig. 2A). To investigate whether specific CNVs contribute to the increase in POLE2 expression, the POLE2 mRNA expression analysis was performed across different CNV categories, demonstrating significant differences (Fig. 2B). Tumors with 1-copy deletion showed significantly lower POLE2 expression compared to tumors with amplification or high amplification. Similarly, tumors with no CNV changes exhibited significantly lower POLE2 expression than those with amplification or high amplification. A significant difference was also detected between the amplification and high amplification groups. In contrast, no significant differences were observed between tumors with two-copy deletions and other categories, likely due to the very small sample size. However, since tumors with one-copy deletion and tumors without CNV changes did not show significant differences in POLE2 expression, these results suggest that while CNV amplification is associated with increased POLE2 expression, copy-number loss or stability alone does not fully account for POLE2 expression variability.

**Figure 2.**
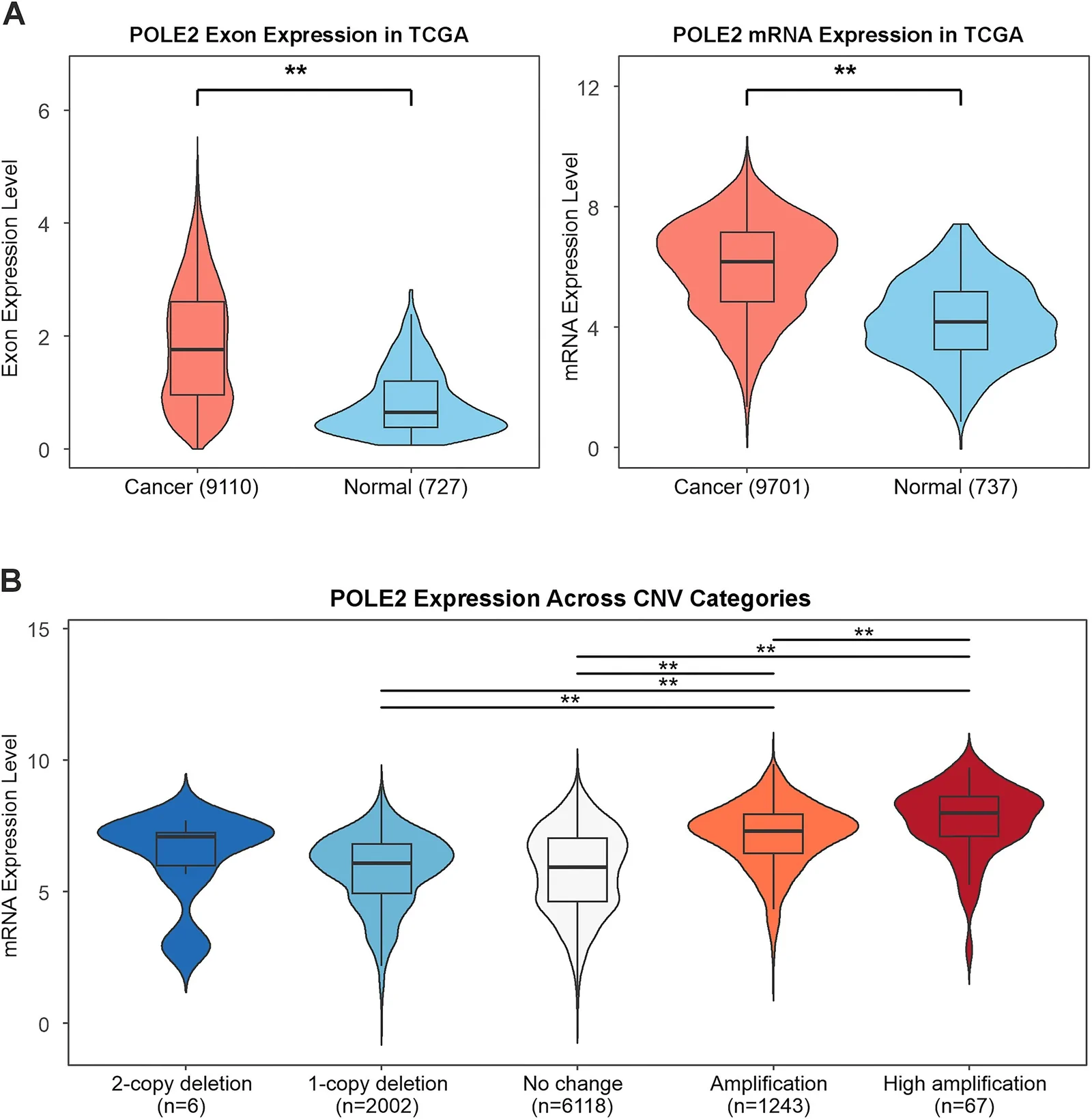
POLE2 expression in cancer and normal tissues based on TCGA data. (**A**) POLE2 exon-level expression (left) and POLE2 mRNA expression levels (right) across TCGA cancer samples and normal tissue samples. Expression distributions are shown as violin plots with embedded boxplots. (**B**) POLE2 mRNA expression levels across CNV categories from TCGA data, where “Amplification” means one-copy gain and “High amplification” means two or more copy gains. Statistical significance was assessed using the Wilcoxon rank-sum test (Mann–Whitney U test). Significance thresholds: *P* ≤ .01 (**), *P* ≤ .05 (*).

To further investigate the relationship between POLE2 expression and CNV, a correlation analysis was performed separately for cancer and normal samples (Supplementary Fig. S3). A weak but statistically significant positive correlation was observed in both groups, with a Pearson correlation coefficient of *r* = 0.22 (*P* = 1.48e-102) in cancer samples and *r* = 0.25 (*P* = 2.28e-10) in normal samples (Supplementary Fig. S3). These findings support that while CNV contributes to POLE2 expression levels, additional regulatory factors are likely to be involved.

To investigate POLE2 expression changes in individual cancer types, we collected data on significant expression differences between cancer and normal tissues from several public databases, including UCSC Xena, GEPIA2 (with and without GTEx), ENCORI, UALCAN, and TIMER. A heatmap summarizing statistically significant differences in POLE2 mRNA expression between cancer and normal samples for each cancer type, as reported by individual databases, is shown in Fig. 3A. The comparison revealed that eight cancer types—BLCA, BRCA, ESCA, HNSC, LUAD, LUSC, STAD, and UCEC—showed consistent significant overexpression of POLE2 in all six databases. Additionally, CESC, COAD, and LIHC demonstrated associations in 5 out of 6 databases; KICH, KIRC, KIRP, READ, and THCA showed associations in 4 out of 6 databases; GBM, CHOL, PAAD, and PRAD showed associations in 3 out of 6 databases. For the eight cancer types where POLE2 overexpression was consistently confirmed across all databases, the expression levels in cancer versus normal tissues are visualized in Fig. 3B. In all cases, POLE2 expression was significantly higher in cancer tissues compared to corresponding normal tissues.

**Figure 3.**
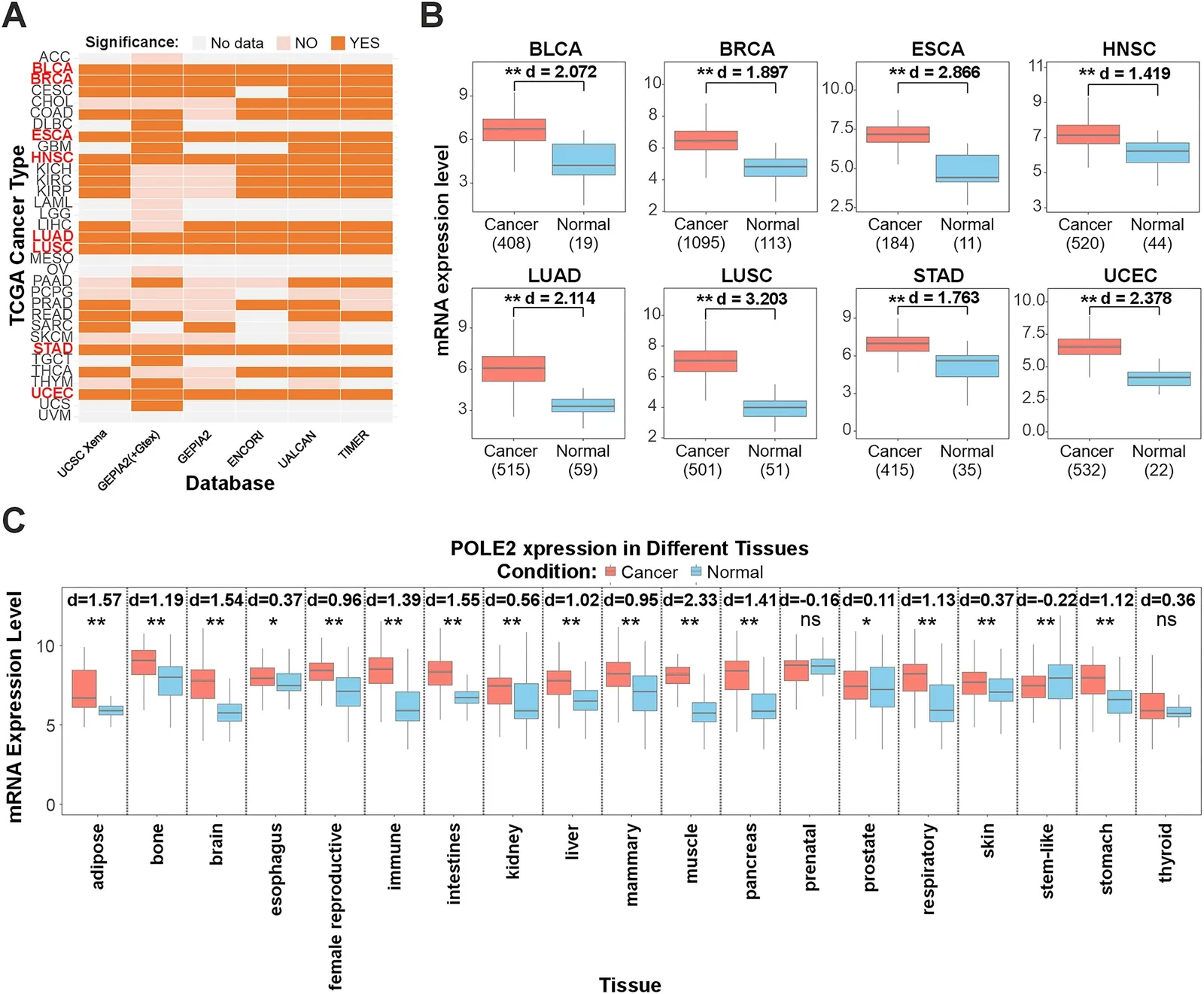
POLE2 mRNA expression in cancer versus normal tissues across databases and tissue types. (**A**) Heatmap summarizing the statistical significance of POLE2 mRNA level differences between cancer and normal samples across multiple independent databases, including UCSC Xena, GEPIA2, GEPIA (+GTEx), ENCORI, UALCAN, and TIMER. Cancer types highlighted in red consistently showed significant differences across all platforms. (**B**) Boxplots of POLE2 expression based on TCGA RNA-seq data for selected cancer types with consistent significance across databases shown in panel A. Each plot displays the standardized mean difference (Cohen’s *d*). (**C**) POLE2 mRNA expression levels across different tissues from ARCHS4 human RNA-seq data, comparing cancer (salmon) and normal (skyblue) samples. Significance thresholds: *P* ≤ .01 (**), *P* ≤ .05 (*)

To evaluate the magnitude of expression differences between cancer and normal tissues, Cohen’s d effect sizes were calculated for each cancer type. All comparisons showed large effect sizes (*d* > 1.4), indicating substantial separation between groups. The strongest effects were observed in LUSC (*d* = 3.203), ESCA (*d* = 2.866), and UCEC (*d* = 2.378), suggesting highly consistent and pronounced overexpression of POLE2 in cancer samples relative to normal tissue. LUAD (*d* = 2.114), BLCA (*d* = 2.072), and BRCA (*d* = 1.897) also showed strong effects, while STAD (*d* = 1.763) and HNSC (*d* = 1.419), though slightly lower, still met the threshold for a large effect size, which is *d* > 0.8.

Additional cancers with significant POLE2 expression differences in at least three databases are shown in Supplementary Fig. S2. The most pronounced effect was observed in CESC (*d* = 5.421), though this finding is based on only three normal samples and should be interpreted cautiously. Notably strong group separation was also present in KIRP (*d* = 1.851), LIHC (*d* = 1.437), KIRC (*d* = 1.497), and COAD (*d* = 1.397), all showing large standardized effect sizes. Cancers such as KICH (*d* = 1.098), PAAD (*d* = 1.046), READ (*d* = 1.045), and THCA (*d* = 0.945) also demonstrated consistent overexpression of POLE2, while PRAD showed a moderate effect (*d* = 0.554). These findings underscore the consistent and often substantial upregulation of POLE2 in a broad range of tumor types. Consistent with our results, previous studies have reported POLE2 upregulation in specific cancers such as gastric cancer [25]—corresponding well with our STAD findings—as well as in BLCA [26], renal cell carcinoma (including KIRC) [21], and OS [56]. These external data further strengthen the notion that POLE2 may play a broader role across diverse tumor types and justify continued investigation into its potential biological functions and clinical relevance.

To investigate POLE2 expression patterns beyond TCGA datasets, tissue data from Expression AnalyzeR, which is based on transcriptomic data from the ARCHS4 database, were utilized. This dataset includes a broader range of samples, covering both cancer and normal tissues, including samples derived from cell lines. The expression levels of POLE2 across different tissue types are visualized in Fig. 3C. A statistically significant difference between cancer and normal tissues was observed for nearly all tissue types, except for prenatal and thyroid tissue. The most substantial difference was observed in muscle tissue (*d* = 2.33), followed by adipose (*d* = 1.57), intestines (*d* = 1.55), brain (*d* = 1.54), pancreas (*d* = 1.41), immune (*d* = 1.39), bone (*d* = 1.19), respiratory (*d* = 1.13), stomach (*d* = 1.12), and liver (*d* = 1.02), all showing large and statistically significant effect sizes (*P* < .01 or *P* < .05). Moderate but significant differences were also seen in female reproductive (*d* = 0.96), mammary (*d* = 0.95), esophagus (*d* = 0.37), skin (*d* = 0.37), thyroid (*d* = 0.36), and prostate (*d* = 0.11). In contrast, prenatal tissue showed a small but significant decrease in POLE2 expression in cancer (*d* = –0.16), while stem-like tissue showed a similar but non-significant reduction (*d* = –0.22). This inverse trend may reflect the naturally high POLE2 expression in normal proliferative or developmentally plastic tissues, such as embryonic and stem-like cell populations, where POLE2 may play essential roles in DNA replication and genome stability maintenance. In such contexts, malignant transformation might not further elevate its expression, or could even reduce it, depending on cellular differentiation status. These findings indicate that while POLE2 is broadly upregulated in cancer across tissue types, the magnitude and direction of change are tissue-dependent and may be influenced by the intrinsic biology of the tissue of origin. Taken together, TCGA and broader ARCHS4 data highlight specific cancer types and tissues where POLE2 upregulation is consistently observed. These patterns suggest that increased POLE2 expression may act as a tissue-specific indicator of malignancy in selected cancer types.

### Differential expression analysis of genes involved in DNA replication and repair pathways

Expression data for a curated set of genes were extracted from the UCSC Xena database to investigate whether specific DNA replication and repair genes are upregulated in particular TCGA cancer types (Fig. 4). These included B-family polymerases such as subunits of Polε (POLE, POLE2–4), polymerase delta (POLD1–4), and polymerase alpha with its primase partners (POLA1, POLA2, PRIM1, PRIM2), which together drive leading and lagging strand synthesis during DNA replication [57]. Additional groups included the X-family polymerases (POLB – Pol β, POLM – Pol μ, POLL – Pol λ), which are specialized for base excision repair (POLB) and non-homologous end joining (POLM, POLL) [58]; the Y-family polymerases (POLH – Pol η, POLI – Pol ι, POLK – Pol κ), which perform translesion synthesis to bypass DNA lesions [59]; and the A-family polymerases (POLQ - Pol θ, POLN - Pol ν, POLG - Pol γ), which participate in mitochondrial DNA replication (POLG), translesion synthesis and base excision repair (POLQ), and error-prone bypass of DNA lesions (POLN) [60]. Only the genes encoding catalytic subunits were included in the analysis for the X-, Y-, and A-family polymerases. Across 20 cancer types, POLE2 stands out as the most overexpressed subunit within the group of replicative polymerases. In contrast, most translesion synthesis and alternative polymerases are downregulated across cancer types. This includes REV3L, POLM, POLL, POLI, POLK, REV1, POLN, and POLG. An exception to this trend is POLQ, which is markedly upregulated despite being a non-replicative polymerase. The consistent upregulation of POLQ presented here aligns well with prior findings [61, 62], reinforcing its emerging role in promoting replication stress tolerance and genomic instability in cancer. Notably, TGCT is the only cancer type showing downregulation of POLQ, and it displays particularly strong suppression of POLN, POLI, and POLK. This expression pattern aligns with prior reports indicating that TGCT cells are deficient in key DNA repair pathways, particularly mismatch repair [63] and nucleotide excision repair, including interstrand crosslink repair [64].

**Figure 4.**
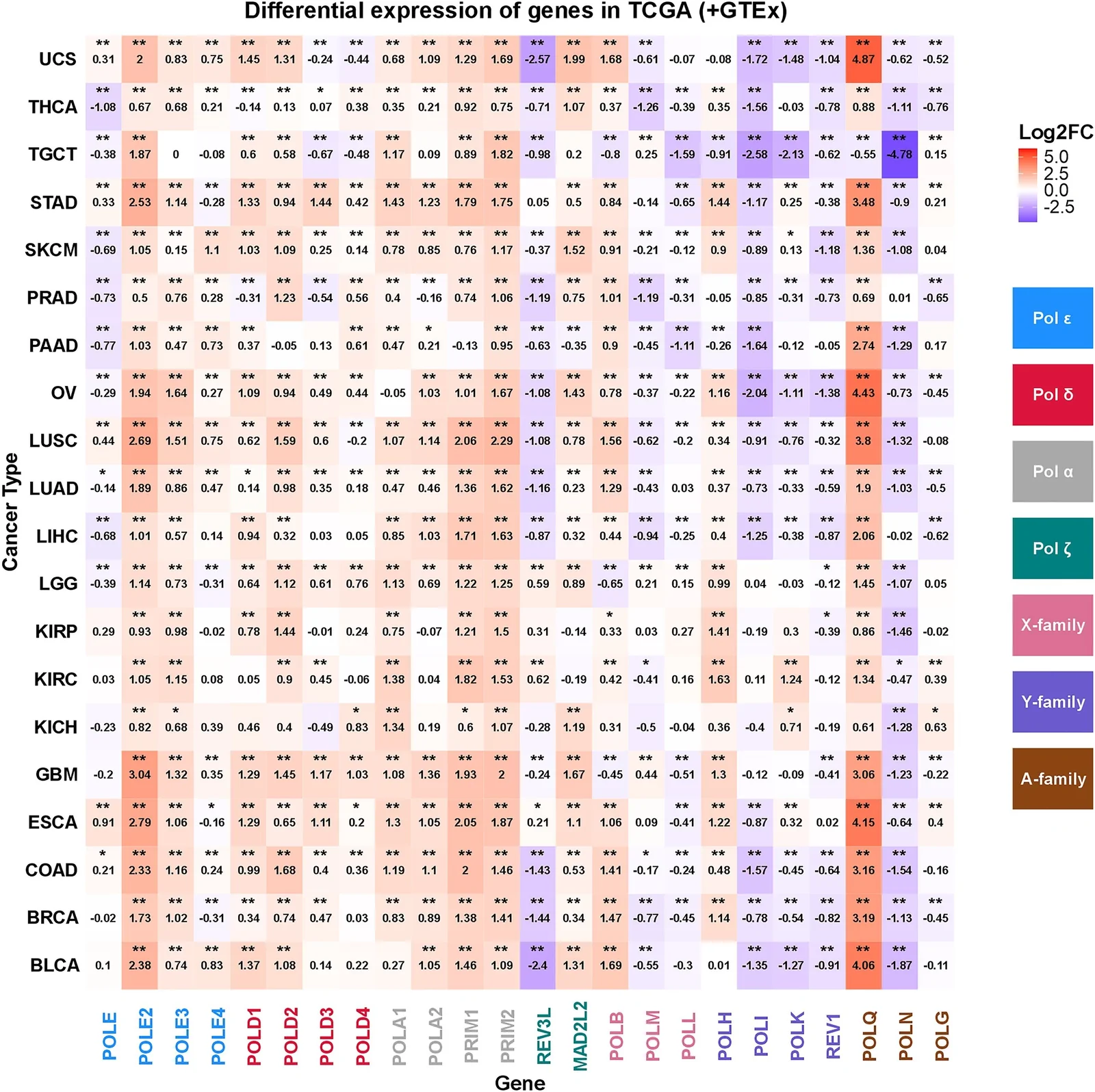
Differential gene expression analysis of selected DNA replication and repair genes using combined TCGA and GTEx data. The heatmap displays the exact log2 fold change (Log2FC) values alongside relative expression shading for each cancer type. Statistical significance is indicated by asterisks, and the colored blocks on the right categorize genes into their respective families. Significance thresholds: *P* ≤ .01 (**), *P* ≤ .05 (*)

Our findings are consistent with earlier reports showing CMG-Polε gene expression profiles. CDC45, for instance, has been shown to be markedly upregulated in various cancers, including thyroid cancer [65], colorectal cancer [66], non-small cell lung cancer [67], esophageal squamous cell carcinoma [68], hepatocellular carcinoma [69], and kidney renal clear cell carcinoma [70]. 

Likewise, MCM subunits, particularly MCM2, MCM4, MCM5, and MCM7, have been repeatedly reported as overexpressed across diverse tumor types such as breast, lung, gastric, colorectal, ovarian, oral squamous cell carcinoma, and hepatocellular carcinoma [71, 72]. These findings have been validated across multiple ethnic cohorts and sample sizes, further supporting the widespread dysregulation of helicase components in tumorigenesis.

The GINS complex, essential for helicase activation, also shows frequent overexpression, with GINS1, GINS2, and GINS4 notably upregulated in sarcoma [73, 74], glioma [75], breast [76], and thyroid cancer [77].

Experimental data on the expression of TLS and alternative polymerases remain limited. Contrary to our findings, POLL was reported to be upregulated across multiple cancer types [78]. Notably, that study also documented increased expression of replicative polymerases, including POLA, POLD, and POLE, and overexpression of POLB—findings that align with our observations. Furthermore, it reported predominant underexpression of POLI and POLK, which is consistent with our results.

There is abundant experimental evidence in the literature regarding the overexpression of POLE2 in most tumors, as previously mentioned in this study. Overall, POLE2 emerges as the most consistently upregulated polymerase subunit across 20 TCGA cancer types. The reason for POLE2 upregulation remains unclear. As it lacks catalytic activity and other polymerase subunits are not similarly overexpressed, its role in cancer may not be limited to DNA replication. Components of the replicative helicase, including CDC45, MCMs, and GINS subunits, also show recurrent overexpression. In contrast, translesion and several non-replicative polymerases are broadly downregulated in tumors, except for POLQ, whose strong upregulation suggests a specialized role in the replication stress response.

### POLE2 expression association with tumor progression

To investigate whether POLE2 expression in cancer tissues varies across cancer stages, POLE2 expression data from TCGA were obtained through the UCSC Xena platform and cBioPortal. Among 21 cancer types from UCSC data, only four—namely ACC (*P* = .0154), ESCA (*P* = .0388), LUSC (*P* = .0014), and TGCT (*P* = .0102)—exhibited significant changes in POLE2 mRNA levels across stages (Fig. 5A). In the cBioPortal dataset, the Non-Small Cell Lung Cancer category comprises LUAD and LUSC, while Renal Non-Clear Cell Carcinoma represents two types of TCGA cancers: KIRP and KICH. Statistical analysis of data for 19 cancer groups from cBioPortal identified seven types with significant stage-related variation: ACC (*P* = .00286), KIRP/KICH (*P* = .0349), KIRC (*P* = .00187), BRCA (*P* = .000856), LUAD/LUSC (*P* = 4.64 × 10^−6^), TGCT (*P* = .00351), and CESC (*P* = .00458) (Fig. 5B). To determine more specifically which stages have significantly different POLE2 expression, a pairwise comparison between stages was performed (Fig. 5).

**Figure 5.**
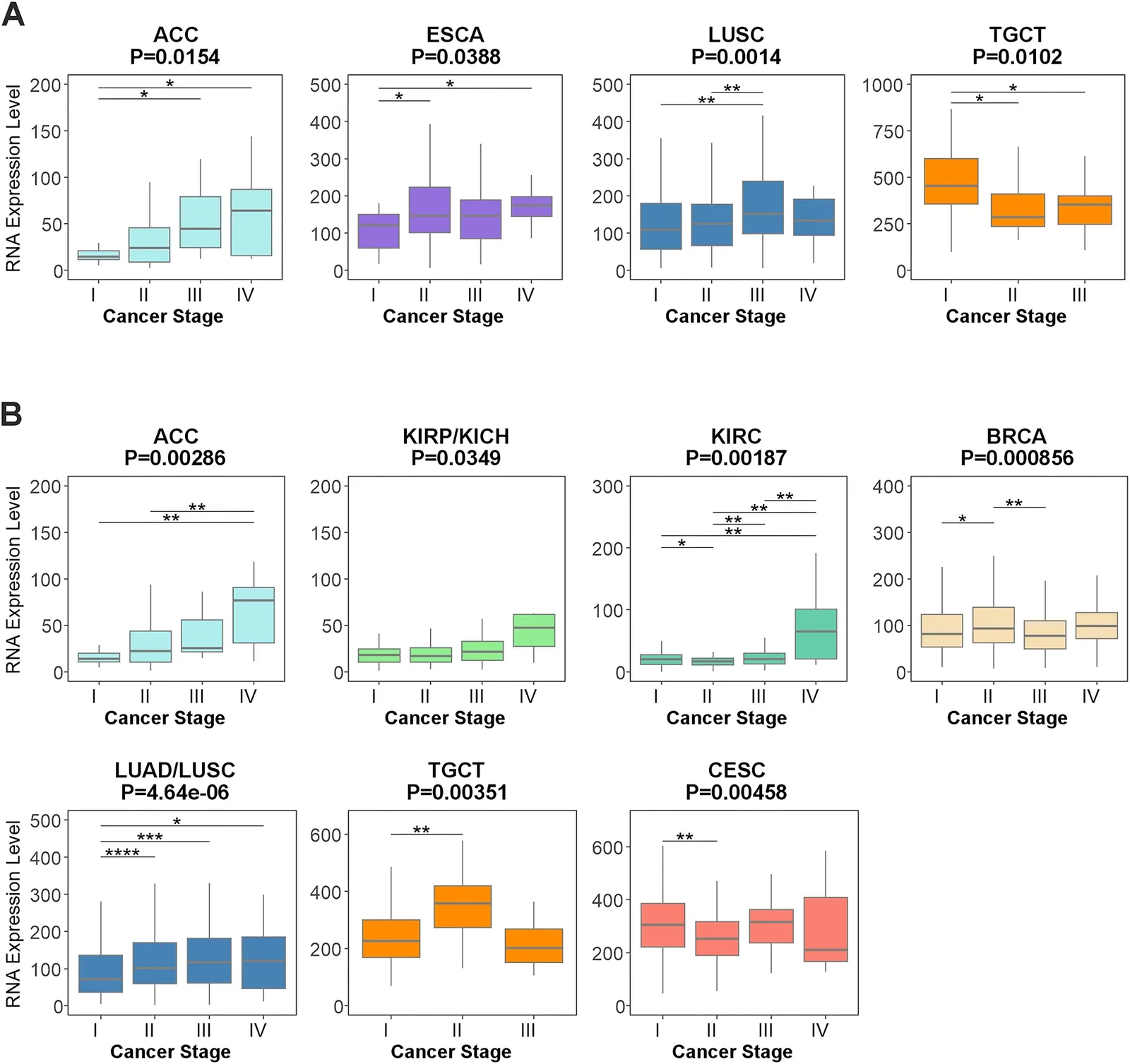
POLE2 mRNA expression levels across cancer stages in selected tumor types. (**A**) Batch effect–normalized RNA-seq expression (RSEM) data from the UCSC Xena platform. (**B**) Batch effect–normalized RNA-seq expression (RSEM) data from cBioPortal. The cancers shown exhibited statistically significant differences in POLE2 expression across clinical stages. Boxplots display RNA expression levels across stages I–IV. Overall significance was assessed using the Kruskal–Wallis test, followed by pairwise Wilcoxon rank-sum tests for post hoc comparisons. *P*-values were adjusted using the Benjamini–Hochberg method to control for multiple testing. Significance thresholds: *P* ≤ .01 (**), *P* ≤ .05 (*).

ACC, KIRP, and KIRC display a clear upward trend in POLE2 expression across cancer stages. In ACC, expression increases significantly between stages I and III, and stages I and IV, with a stronger confirmation of this trend in the cBioPortal dataset. KIRC shows consistent and pronounced increases in expression, with significant differences observed between almost all adjacent stages, highlighting a progressive upregulation as the cancer advances. KIRP demonstrates a milder upward trend, with statistically significant differences overall, although changes between individual stages are less pronounced. Previous work based on larger renal cell carcinoma cohorts has already shown that POLE2 expression rises with tumor progression [21]. However, the role of POLE2 expression in ACC progression was not precisely highlighted. Nevertheless, given that this previous study showed that POLE2 expression consistently correlates with survival, the influence of POLE2 in ACC needs further investigation.

ESCA and LUAD/LUSC exhibit a slight increase in POLE2 expression as cancer progresses. In ESCA, significant differences are observed between stages I and II, and between stages I and IV, suggesting a modest rise. LUAD/LUSC demonstrates a more apparent stepwise increase, particularly at later stages, with significant differences observed between stage I and all subsequent stages.

LUSC, BRCA, TGCT, and CESC show no strong dependence of POLE2 expression on cancer stage. LUSC shows a gradual increase in POLE2 expression up to stage III, followed by a slight but non-significant decrease at stage IV. BRCA similarly shows changes between early and later stages, specifically between stages I and II, and I and IV, but overall, the progression is modest. In TGCT, according to the UCSC Xena database, POLE2 expression was highest in stage I cancers, while data from cBioPortal showed this for stage II, indicating inconsistent results. CESC shows a difference between stages only between stages I and II, which is insufficient to conclude a clear trend.

Overall, POLE2 expression shows cancer-type-specific stage dependence. In cancers such as ACC, KIRC, and KIRP, expression tends to increase progressively with advancing stage, suggesting a potential link to tumor progression. ESCA and LUAD/LUSC exhibit a modest but noticeable increase in expression across stages. In contrast, cancers like TGCT display an apparent decrease in POLE2 levels across stages, while others, including LUSC, BRCA, LUAD/LUSC, and CESC, show no consistent trend. These findings indicate that the relationship between POLE2 expression and cancer stage is not universal but may reflect distinct biological roles of POLE2 in different tumor types.

### POLE2 promoter methylation analysis

One of the ways to investigate gene expression activity is to look at the methylation level of the gene promoter. We used HumanMethylation450 BeadChip data, which targets 96% of CpG islands in the human genome [79], that were part of the TCGA project. Combined methylation levels of all CpG sites (Fig. 6A) revealed that the POLE2 promoter is significantly hypermethylated (Δβ = 0.024) in cancer samples (*n* = 8281) compared with normal samples (*n* = 723). Upstream of POLE2, there are 12 CpG cites: cg11095277, cg10038907, cg09325174, cg10195098, cg26030540, cg15522118, cg15455399, cg23418097, cg23761011, cg04379738, cg27022570, and cg3496872 (Fig. 6B). If we analyze the methylation level of each CpG site separately, we see that a predominant part of them is hypermethylated, while hypomethylation is observed in only two sites. Among the hypermethylated CpG sites, cg04379738 demonstrated the most substantial methylation difference between cancer and normal tissues (Δβ = 0.107), indicating meaningful hypermethylation in cancer. The differences at all other sites were below 0.1 and therefore are not considered biologically meaningful despite being statistically significant (Fig. 6B). CpG sites cg11095277 (Δβ = –0.008) and cg3493872 (Δβ = –0.021) showed hypomethylation in cancer, but the differences were below 0.1 and therefore not biologically meaningful. Additionally, these two sites are not located within CpG island 52, based on data from the UCSC Genome Browser (Fig. 6C), which can mean that they are less associated with the POLE2 promoter region. Although the POLE2 promoter has not been definitively characterized, the “EPDnew v6” track [80] in the UCSC Genome Browser identifies an experimentally supported promoter, POLE2_1 (chr14:50154922–50154981, 60 bp in length). In contrast, the “Genome Segmentation by combined Segway + ChromHMM from the ENCODE/Analysis” track [81] defines broader regulatory regions, including a TSS region (Predicted promoter region including transcription start site) and a PF (Predicted promoter flanking region) region. These span from chr14:50 152 604 to chr14:50 155 774 across various cell lines, covering a 3170 bp fragment. However, this extended region does not include the CpG sites cg11095277 and cg3493872; therefore, these sites were excluded from the promoter region in further analyses.

**Figure 6.**
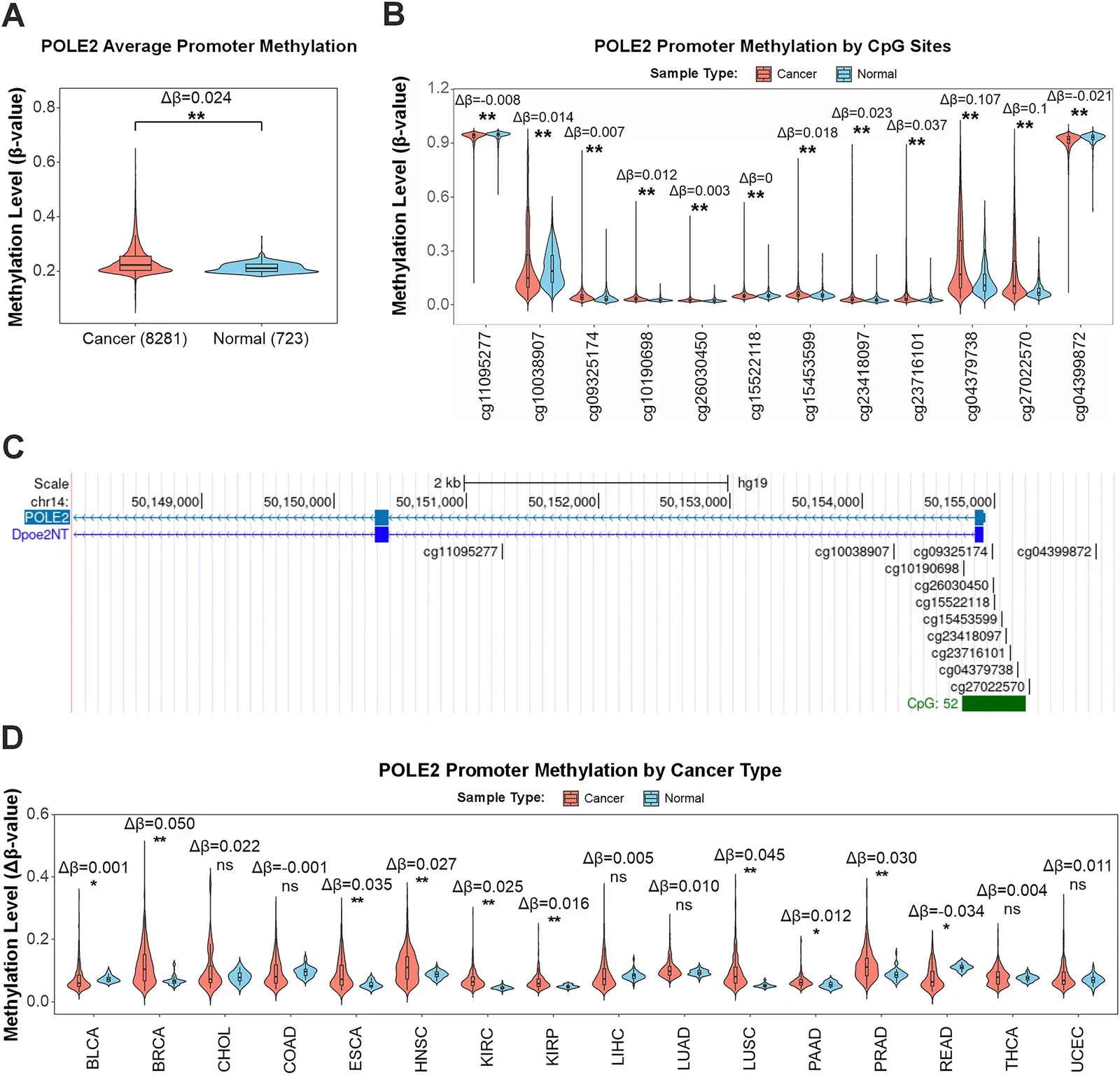
Comparison of POLE2 CpG site methylation levels in cancer and normal samples based on Illumina HumanMethylation450 BeadChip (450K array) data. (**A**) Average POLE2 methylation levels across TCGA cancer samples (*n* = 8281) and TCGA normal samples (*n* = 723). (**B**) Methylation levels at individual CpG sites within the POLE2 gene region, shown separately for cancer and normal tissues. Numbers represent mean methylation difference (Δβ)—the difference in average β-values between cancer and normal groups. (**C**) UCSC Genome Browser view of the POLE2 locus, with CpG probe locations from the 450K methylation track. (**D**) Average POLE2 promoter methylation levels for CpG sites within the POLE2 promoter region. Statistical significance was assessed using the Wilcoxon rank-sum test (Mann–Whitney U test). Significance thresholds: *P* ≤ .01 (**), *P* ≤ .05 (*).

Next, POLE2 promoter methylation levels were assessed for 16 TCGA cancer types (Fig.6D). Statistically significant increases in methylation were observed in BRCA (Δβ = 0.050), ESCA (Δβ = 0.035), HNSC (Δβ = 0.027), KIRC (Δβ = 0.025), KIRP (Δβ = 0.016), LUSC (Δβ = 0.045), PAAD (Δβ = 0.012), PRAD (Δβ = 0.030), BLCA (Δβ = 0.001), and READ (Δβ = –0.034). However, the extent of these differences remained modest across all cases and is unlikely to reflect a substantial biological impact. In contrast, CHOL (Δβ = 0.022), COAD (Δβ = –0.001), LIHC (Δβ = 0.005), LUAD (Δβ = 0.010), THCA (Δβ = 0.004), and UCEC (Δβ = 0.011) showed no statistically significant differences in methylation between cancer and normal samples. Given that overall differences in promoter methylation are minimal—even among statistically significant cases—it is unlikely that epigenetic regulation through DNA methylation is a major contributor to the upregulation of POLE2 mRNA expression in cancer. Nevertheless, the promoter region is predominantly hypermethylated in cancer samples. To further investigate the potential relationship between methylation and gene expression, we performed Pearson correlation analysis between POLE2 promoter methylation and mRNA expression levels (Supplementary Fig. S4). This analysis revealed a weak but statistically significant negative correlation in cancer samples (*r* = –0.20), indicating that higher methylation is modestly associated with lower POLE2 expression. In contrast, the correlation in normal samples was very weak (*r* = –0.06) and only marginally significant, suggesting that DNA methylation likely plays a limited regulatory role in controlling POLE2 expression.

The correlation between methylation and expression was calculated separately for each cancer to investigate whether this effect is consistent across cancer types. Several tumor types showed notable correlations. TGCT exhibited the strongest negative correlation (*r* = –0.49), followed by LIHC (*r* = –0.24) and CESC (*r* = –0.13), indicating that increased methylation is associated with reduced POLE2 expression in these cancers. In contrast, LUSC (*r* = 0.12) and ESCA (*r* = 0.16) showed positive correlations, suggesting that the relationship may vary across cancer types and is not strictly repressive (Supplementary Fig. S4). In normal adjacent tissues, fewer cancer types showed significant correlations, likely due to smaller sample sizes. Still, positive correlations were observed in BRCA (*r* = 0.24), LIHC (*r* = 0.55), and BLCA (*r* = 0.63).

In summary, the mean difference in promoter methylation between cancer and normal tissues is generally slight. However, the consistent hypermethylation observed across most CpG sites and in average comparisons suggests the involvement of additional regulatory mechanisms in POLE2 promoter control or potential inaccuracies in promoter region annotation. A positive correlation between promoter methylation and mRNA expression in normal tissues may reflect non-canonical regulation, such as activity from non-CGI (non- CpG island) or alternative promoters where methylation does not repress transcription. A weaker positive correlation in cancer may indicate disrupted epigenetic control or partial loss of regulatory stability [82].

### Isoform expression analysis

Alternative splicing (AS) generates multiple mRNA isoforms from a single gene, which are often regulated in a tissue- and cancer-type-specific manner. Many cancer-associated isoforms arise due to aberrant splicing, some of which are unique to tumors. Because of this specificity, isoform-level expression can serve as a biomarker for cancer diagnosis, prognosis, and treatment selection more precisely than total gene expression [83]. Here, we studied transcript abundance relative to the total POLE2 expression (Fig. 7A and Supplementary Fig. S5) and their structure (Fig.7B). Notably, the non-coding isoform POLE2-010 (7 exons) emerges as the most abundant isoform, representing ~32% of total POLE2 abundance in cancer samples, and similarly high proportions in Normal_GTEX (36%) and Normal_TCGA tissues (29%). This high abundance of a non-coding isoform is particularly noteworthy, suggesting a significant, potentially regulatory role despite lacking protein-coding capacity. Among coding isoforms, POLE2-001 (19 exons) is highly represented, comprising around 28% of total POLE2 abundance in cancer tissues and 31% in Normal_GTEX and 35% in Normal_TCGA. Another coding isoform, POLE2-012 (17 exons), also shows significant abundance, consistently around 25% across cancer and normal samples, highlighting stability in its relative expression. Moderately abundant coding isoforms include POLE2-008 (3 exons) and POLE2-002 (18 exons). Non-coding isoforms POLE2-003, POLE2-006, and POLE2-007 (3 exons), as well as problematic isoforms POLE2-004 (2 exons), POLE2-005 (3 exons), and POLE2-011 (4 exons), show reduced abundance in cancer tissues relative to normal tissues. However, detailed isoform-focused studies are necessary to validate this pattern.

**Figure 7.**
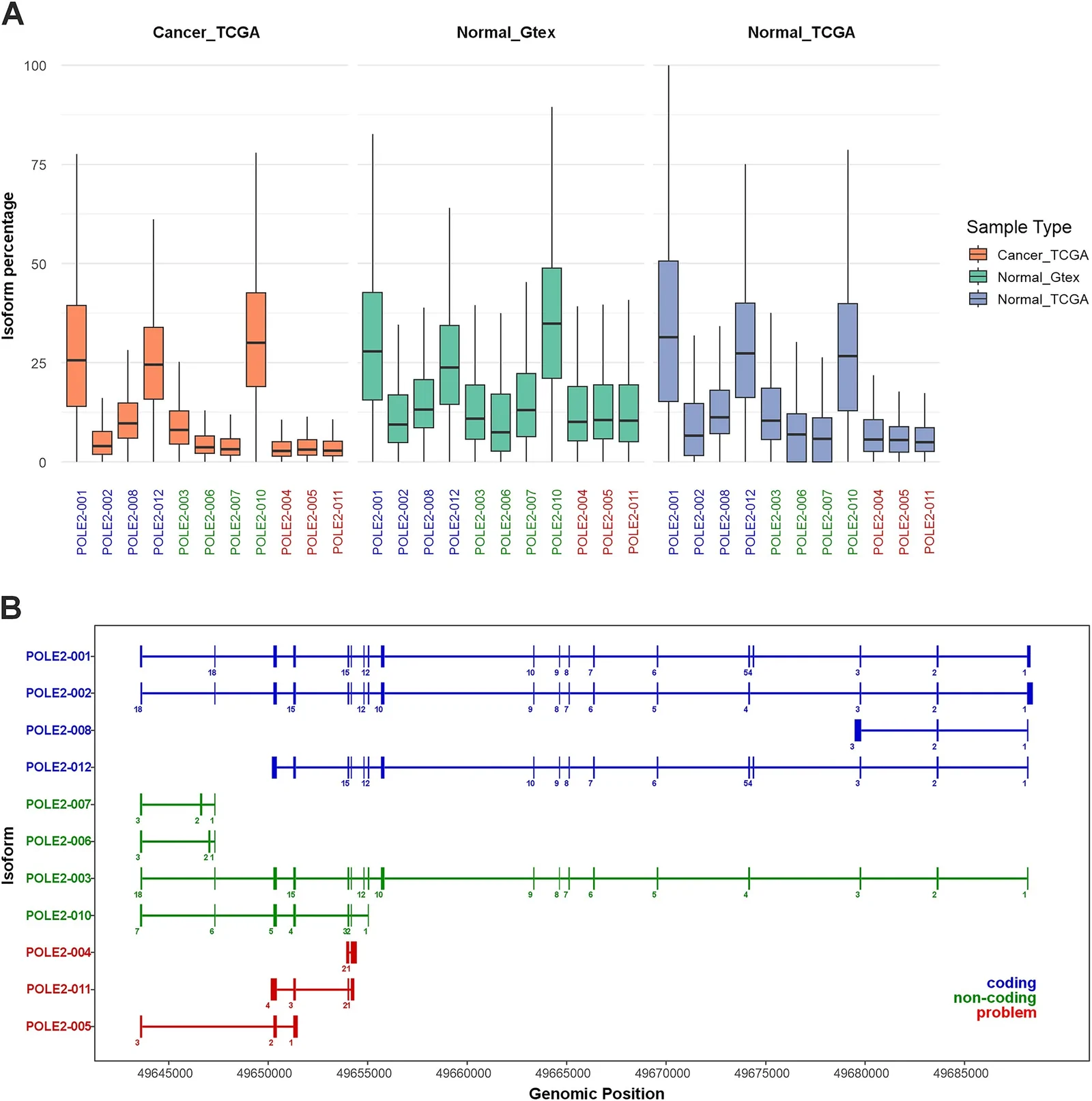
POLE2 isoform expression and transcript structure in cancer and normal tissues. (**A**) Isoform percentage (IsoPct) of each POLE2 transcript across three sample types: cancer (*n* = 9163), GTEx normal (*n* = 5 354), and TCGA normal (*n* = 712), derived from TOIL-processed isoform expression data obtained via the UCSC Xena database. IsoPct represents the proportion of a transcript’s abundance relative to the total expression of its parent gene (POLE2), allowing us to assess isoform usage rather than absolute expression levels. (**B**) Exon–intron structures of 11 POLE2 transcript isoforms aligned to the same genomic locus, based on UCSC Genome Browser annotations. Each horizontal line represents a transcript; vertical blocks indicate exons, and connecting lines represent introns. Isoforms are color-coded by classification: coding (blue), non-coding (green), and problematic/incomplete annotations (red). Significance thresholds: *P* ≤ .01 (**), *P* ≤ .05 (*).

The analysis of POLE2 isoform percentages indicates that the previously observed overexpression of the POLE2 gene in cancer primarily results from increased abundance of two coding isoforms (POLE2-001 and POLE2-012) and one prominent non-coding isoform (POLE2-010). Given that total isoform percentages sum to 100%, the decreased relative proportions of problematic and other less abundant isoforms in cancer tissues likely reflect their dilution due to the substantial upregulation of these dominant isoforms, rather than an actual decrease in their absolute expression levels. This highlights that isoform-specific regulation contributes significantly to the elevated expression of POLE2 observed in cancer.

Focusing on cancer-specific isoforms can enhance diagnostic precision, and we have found that coding POLE2-001 and POLE2-012, as well as non-coding POLE2-010, are predominantly expressed.

Given this significant contribution of both coding and abundantly expressed noncoding isoforms to the overall cancer profile, it is crucial to dissect their specific impact on patient outcomes. Categorizing the transcriptomic data into three distinct functional classes (coding, noncoding, and problematic) reveals a cancer-specific prognostic landscape when evaluating OS (Table 3). The noncoding isoforms demonstrate substantial and independent prognostic value in specific contexts. In the LUAD cohort, high expression of noncoding isoforms is significantly associated with worse OS in both univariate (HR = 1.64; *P* < .001) and multivariate analyses (HR = 1.71; *P* < .001). Similarly, in the MESO cohort, noncoding isoforms retain their significance across both univariate (HR = 1.8; *P* = .015) and multivariate (HR = 2.21; *P* = .011) models. However, these noncoding transcripts completely lack prognostic significance in the ACC and LGG cohorts.

**Table 3.** Univariate and multivariate Cox regression analyses of coding, non-coding, and problematic POLE2 isoform expression for OS in ACC, LGG, LUAD, and MESO cohorts

Characteristic	Cohort distribution	Univariate analysis			Multivariate analysis		
		HR	95% CI	*P*-value	HR	95% CI	*P*-value
**ACC**	***N* = 59**						
coding isoform expression high/low	30 / 29	1.79	0.77-4.14	0.2	1.14	0.46-2.84	0.8
non-coding isoform expression high/low	30 / 29	1.72	0.75-3.94	0.2	1.24	0.43-3.56	0.7
problematic isoform expression high/low	30 / 29	1.11	0.49-2.54	0.8	0.67	0.24-1.85	0.4
**LGG**	***N* = 502**						
coding isoform expression high/low	251 / 251	1.81	1.25-2.63	**0.002**	1.19	0.80-1.77	0.4
non-coding isoform expression high/low	251 / 251	1.16	0.81-1.66	0.4	0.72	0.49-1.06	0.10
problematic isoform expression high/low	250 / 252	1.25	0.86-1.80	0.2	1.31	0.90-1.90	0.2
**LUAD**	***N* = 534**						
coding isoform expression high/low	267 / 267	1.34	1.01-1.78	**0.041**	1.29	0.97-1.73	0.084
non-coding isoform expression high/low	267 / 267	1.64	1.24-2.19	**<0.001**	1.71	1.28-2.28	**<0.001**
problematic isoform expression high/low	267 / 267	1.05	0.79-1.39	0.7	1.15	0.87-1.54	0.3
**MESO**	***N* = 87**						
coding isoform expression high/low	44 / 43	1.65	1.04-2.62	**0.035**	2.02	1.21-3.40	**0.008**
non-coding isoform expression high/low	44 / 43	1.80	1.12-2.89	**0.015**	2.21	1.20-4.07	**0.011**
problematic isoform expression high/low	44 / 43	0.72	0.45-1.15	0.2	0.99	0.53-1.84	>0.9

Abbreviations: CI = confidence interval, HR = hazard ratio.

The prognostic behavior of the coding isoforms is equally variable. They serve as independent predictors of poor survival solely in the MESO cohort across both univariate (HR = 1.65; *P* = .035) and multivariate (HR = 2.02; *P* = .008) analyses. In the LGG cohort, coding isoforms are univariately significant (HR = 1.81; *P* = .002), but this significance collapses in the multivariate model (*P* = .4). A similar trend occurs in the LUAD cohort, where high coding isoform expression associates with poor prognosis in the univariate model (HR = 1.34; *P* = .041) but appears not to be significant during multivariate adjustment (*P* = .084). Furthermore, coding isoforms exhibit no significance whatsoever in the ACC cohort. Finally, across all four datasets, problematic isoforms consistently fail to demonstrate any significant association with OS in either univariate or multivariate models.

When we align these isolated transcript results with the prior total POLE2 survival data, the specific RNA variants responsible for patient mortality become clearly identifiable. In the LUAD cohort, while total POLE2 expression was a significant independent predictor of poor survival, the isoform analysis reveals this signal is overwhelmingly driven by the noncoding transcripts. For the MESO cohort, the robust multivariate significance previously seen with total POLE2 is supported by a dual contribution from both the coding and noncoding functional classes. Conversely, the LGG cohort perfectly reflects the attenuation seen in the total gene analysis, with coding isoforms losing their initial univariate significance during multivariate modeling and noncoding isoforms lacking significance entirely. Finally, the ACC cohort presents an interesting dilution effect where total POLE2 expression maintained strong independent prognostic significance, but parsing the transcripts into separate functional classes fractures the statistical power, resulting in neither coding nor noncoding isoforms exhibiting significance across the evaluated models.

### POLE2 protein levels in cancer samples do not always correspond to their mRNA expression levels

The comparison between mRNA and protein expression levels across seven cancer types—BRCA, GBM, LIHC, LUAD, LUSC, PAAD, and UCEC—was conducted using data from the UALCAN database (Fig.8). These cancer types were selected because CPTAC mass spectrometry data with profiled POLE2 protein levels were available only for a limited number of cancer types.

**Figure 8.**
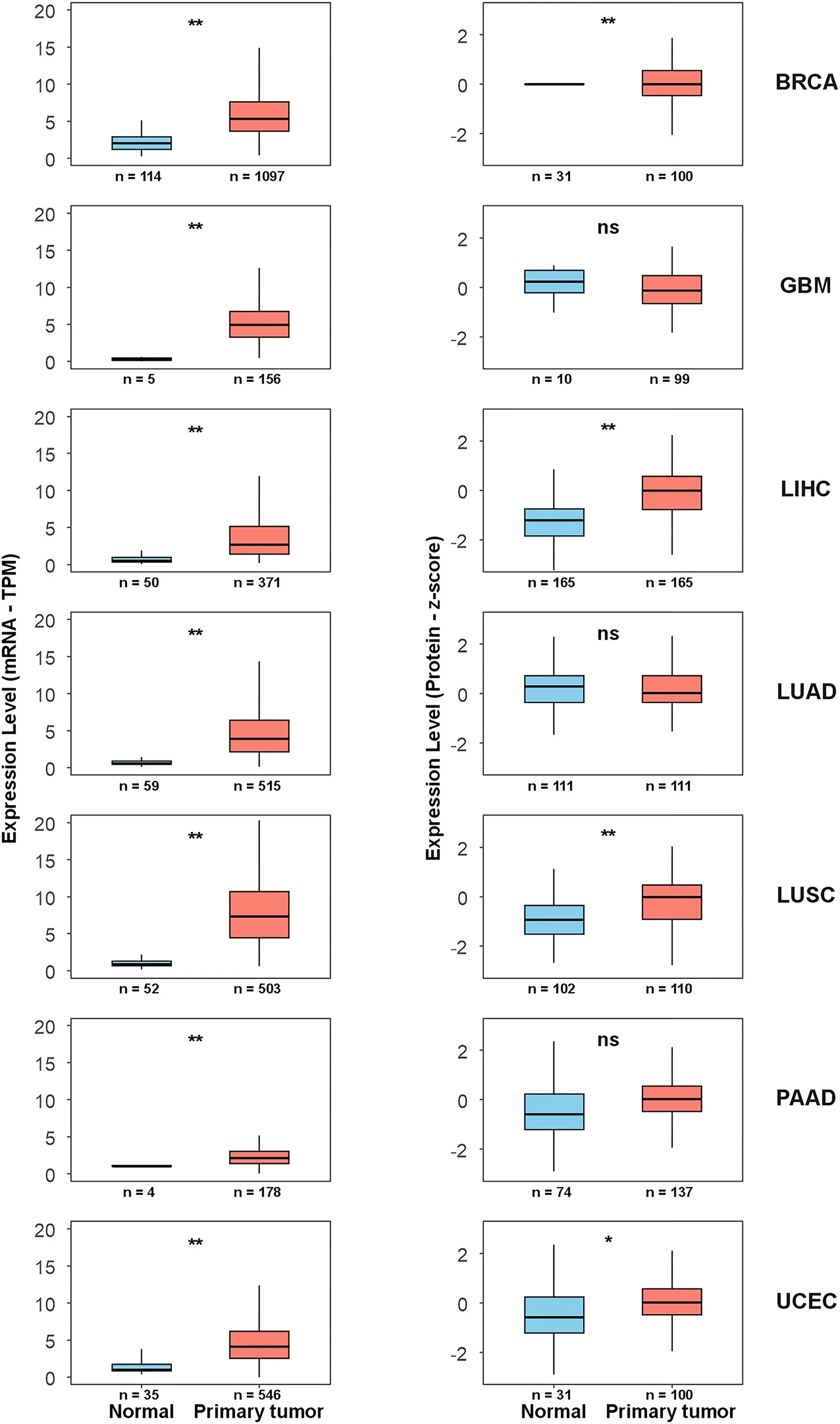
Comparison of POLE2 mRNA and protein expression in cancer versus normal tissues based on the UALCAN database. Boxplots on the left show POLE2 mRNA expression levels (TPM) across normal and primary tumor tissues in selected cancer types. Boxplots on the right represent corresponding POLE2 protein expression levels (*z*-scores) in the same tissue types. Statistical significance was calculated using the Wilcoxon rank-sum test (Mann–Whitney U test). Significance levels: *P* ≤ .01 (**), *P* ≤ .05 (*).

In BRCA, mRNA levels in tumors (*n* = 1097, median = 5.32) were significantly higher than in normal tissues (*n* = 114, median = 2.02), and this difference was also seen at the protein level (tumor *n* = 100, median = 0.24; normal *n* = 31, median = −0.19). GBM showed a strong increase in mRNA (tumor *n* = 156, median = 4.92; normal *n* = 5, median = 0.26), but no significant change in protein (tumor *n* = 99, median = 0.02; normal *n* = 10, median = −0.06). In LIHC, transcript levels rose notably (tumor *n* = 371, median = 5.31; normal *n* = 50, median = 0.35), and protein values also increased (tumor *n* = 99, median = 0.53; normal *n* = 10, median = −0.36). LUAD showed elevated mRNA levels in tumors (*n* = 515, median = 4.97) compared to normal (*n* = 59, median = 0.32), but protein levels remained similar (both groups *n* = 165, medians = 0.08 and 0.03), with no significant difference. In LUSC, mRNA was strongly upregulated (tumor *n* = 503, median = 7.27; normal *n* = 52, median = 0.28), and protein levels also increased (tumor and normal *n* = 111, medians = 0.38 and −0.23). PAAD displayed a rise in transcript levels (tumor *n* = 178, median = 1.05; normal *n* = 4, median = 1.07), though the difference was small, and protein values showed no significant variation (tumor *n* = 137, median = 0.12; normal *n* = 74, median = −0.10). In UCEC, transcript levels increased clearly (tumor *n* = 546, median = 4.38; normal *n* = 35, median = 0.56), and protein also showed no significant change (tumor *n* = 100, median = 0.27; normal *n* = 31, median = −0.19).

Across all examined cancers, the magnitude of change in mRNA levels between tumor and normal tissues was consistently greater than that observed at the protein level. BRCA, LIHC, LUSC, and UCEC showed significant changes in both data types. In contrast, GBM, LUAD, and PAAD displayed increased mRNA without significant differences in protein levels. This can be partially explained by the higher abundance of the non-coding POLE2-010 isoform in cancer, as described earlier, as well as translational efficiency, protein stability, and the sensitivity of the mass spectrometry method utilized in that study.

### Mutations in POLE2 identified in cancer samples

POLE2 encodes a core structural subunit of the DNA Polε complex, which is essential for high-fidelity leading-strand DNA synthesis during DNA replication. Although POLE2 lacks enzymatic activity, mutations in this gene can compromise replication accuracy by destabilizing the polymerase complex or impairing its interaction with DNA. Analysis of somatic mutation data from the COSMIC database [84] and previous studies has revealed recurrent POLE2 alterations across several cancer types, including colorectal, endometrial, renal, and biliary tract tumors. These mutations include nonsense variants, frameshift mutations, and missense changes. Supplementary Table S2 and the lollipop plot (Fig. 9) summarize all POLE2 mutations with a recurrence count of >1, curated from the COSMIC database, highlighting their distribution and functional classification across the gene.

**Figure 9.**
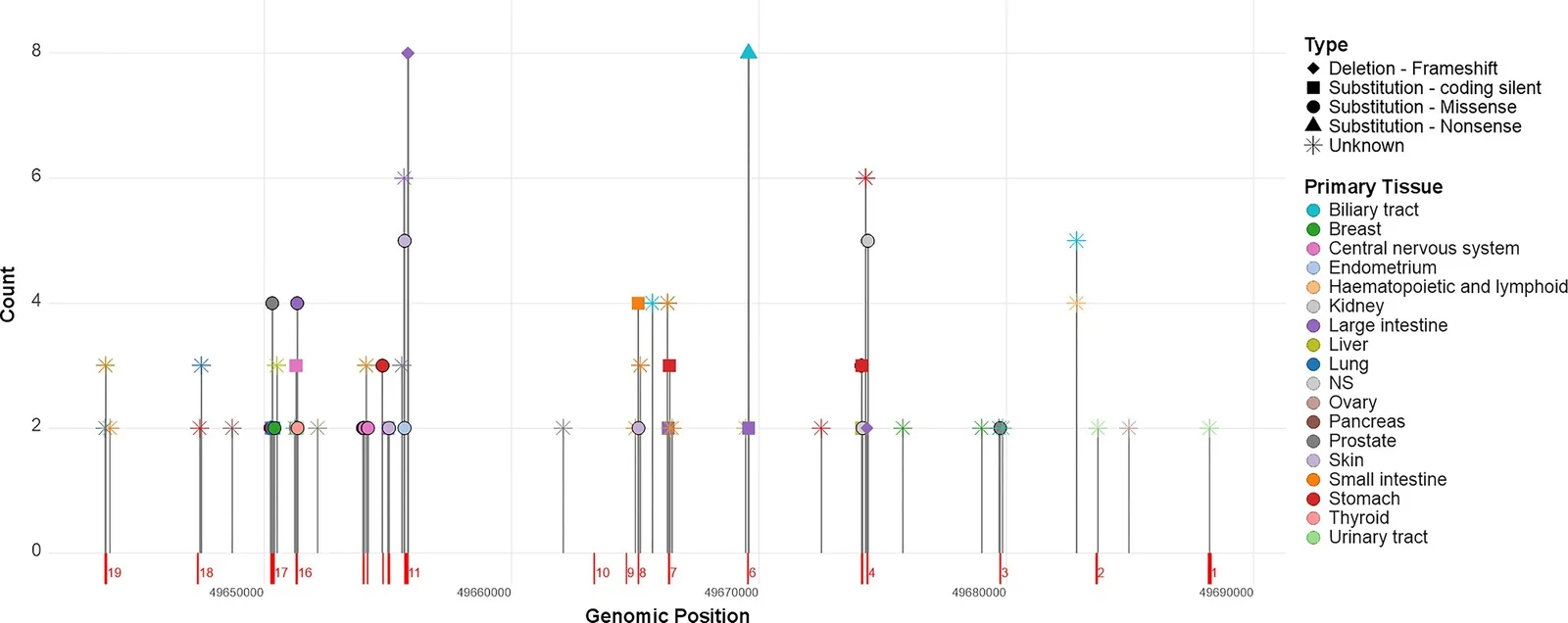
Distribution of POLE2 mutations across exons and introns. Lollipop height indicates mutation frequency in cancer samples. Colors represent tissue of origin, and shapes denote mutation types. Exons are shown as red blocks; introns span between them.

A germline POLE2 variant c.823C > A was reported in five individuals from the same family affected by colorectal cancer, along with a frameshift mutation, c.1328_1329insT (p.Leu443Phefs*17), predicted to disrupt protein function [85]. Subsequently, another frameshift mutation, c.1406dupT (p.Leu469PhefsTer17), was identified in four samples from colorectal cancer patients [86]. Later, additional germline POLE2 variants, including c.655A > G (one heterozygous carrier), c.1414T > C (two heterozygous carriers), c.50G > C (one carrier), and c.856A > G (one carrier), were reported [87]. Although many of these alterations are currently classified as variants of uncertain significance, their recurrence in colorectal cancer cohorts and their potential to impair replication fidelity suggest a possible role in promoting genomic instability and tumor development. Germline mutations found in these studies are summarized in Table 4.

**Table 4.** Germline mutations identified in POLE2

Protein change	Mutation type	Nucleotide change	Tissue and cancer type	Reference
p.R17P	Substitution - Missense	c.50G > C	Colorectal carcinoma	[87]
p.T219A	Substitution - Missense	c.655A > G	Colorectal carcinoma	[87]
p.M286V	Substitution - Missense	c.856A > G	Colorectal carcinoma	[87]
p.L275I	Substitution - Missense	c.823C > A	Colorectal carcinoma	[85]
p.Y472H	Substitution - Missense	c.1414T > C	Colorectal carcinoma	[87]
p.L443Ffs*17	Frameshift- Insertion	c.1328_1329insT	Colorectal carcinoma	[85]
p.L469FfsTer17	Frameshift- duplication	c.1406dupT	Colorectal carcinoma	[86]

To assess the spatial distribution of POLE2 mutations, the protein structure was visualized using the cryo-EM structure available in the Protein Data Bank (PDB ID: 7PFO) [88]. COSMIC-annotated mutations were mapped onto this structure using ChimeraX software. Mutations with a recurrence count greater than four were highlighted to identify regions of potential functional relevance (Supplementary Fig. S6). Notably, a clustering of mutations was observed in a specific region of the protein, suggesting a potential hotspot for structural or functional disruption. This region was marked by a higher local density of missense and truncating mutations. Notably, the number of identified mutations in POLE2 is relatively low compared to the number of identified mutated variants of POLE, the catalytic subunit of DNA polymerase ε [89]. However, this may reflect the significance of functional POLE2 for cell physiology as previously proposed for subunits of the CMG helicase regarded as “engines” critical for tumor cell survival [90].

### Enrichment analysis of POLE2-correlated genes in cancer

In order to investigate molecular mechanisms of POLE2 overexpression and association with cancer, we studied genes that are highly correlated (Pearson correlation value, PCC > 0.8) with POLE2 and therefore have similar expression patterns. To obtain convincing results, we used four databases as a source of POLE2-correlated genes and overlapped them using an UpSet plot.

The UpSet plot (Fig. 10) illustrates how genes are distributed across five datasets: GTEx_Normal and Analyzer_Normal for normal tissue, and GEPIA_Cancer, Analyzer_Cancer, and UALCAN_Cancer for tumor tissue (Fig. 10 and Supplementary Figs S7 and S8). The total number of gene occurrences across all sets is 2303 (Set Size Total), while 1656 genes fall into exclusive intersections (Intersection Size Total), indicating the number of unique genes assigned to specific dataset combinations.

**Figure 10.**
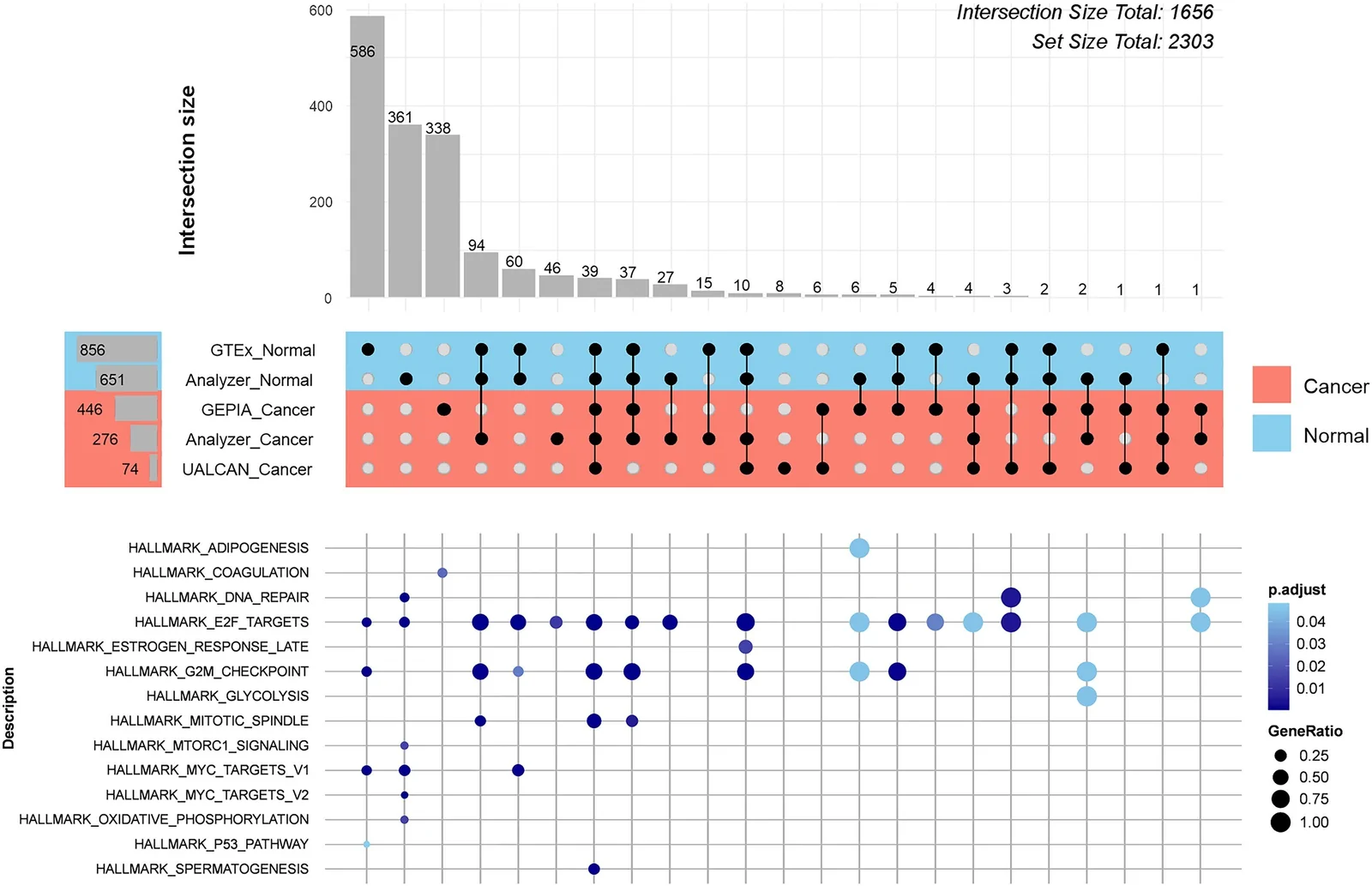
Overlap of genes highly correlated with POLE2 across multiple datasets in cancer and normal tissues. UpSet plot showing the intersection of genes with a Pearson correlation coefficient > 0.8 with POLE2 expression, derived from six datasets from four independent databases: GTEx, GEPIA, UALCAN, and AnalyzeR, each separated by sample type. Top bars indicate the number of overlapping genes shared among dataset combinations, while the left bars show the total number of highly correlated genes per dataset. “Set Size Total” represents the total number of gene occurrences across all input sets, counting duplicates when a gene appears in multiple sets. In contrast, “Intersection Size Total” reflects the number of unique genes that are assigned to exclusive intersections—each gene counted only once based on its specific combination of set membership. Dot plot representing the results of ORA performed on intersecting POLE2-correlated genes using the HALLMARK gene set collection. Dot size reflects the gene ratio, and color intensity indicates statistical significance (adjusted *P*-value).

Cancer-specific exclusive intersections are led by “GEPIA_Cancer,” which alone contains 338 unique genes, making it the largest single-source cancer gene set. Less prevalent cancer-only intersections include “Analyzer_Cancer” with 46 genes and the combined “UALCAN_Cancer.GEPIA_Cancer” with six genes. Functional enrichment analysis of these cancer-specific groups reveals that “GEPIA_Cancer” genes are enriched in the hallmark coagulation pathway (adjusted *P* = .024), suggesting potential links to tumor-associated clotting mechanisms. “Analyzer_Cancer” genes are enriched in E2F targets (*P* = .013), indicating involvement in cell cycle regulation.

For normal-specific intersections, the largest group is “GTEx_Normal” with 586 unique genes, followed by “Analyzer_Normal” with 361 genes, and their shared intersection “Analyzer_Normal.GTEx_Normal” containing 60 genes. These normal cell-related groups are strongly enriched in proliferation-associated pathways. “GTEx_Normal” genes are enriched in G2M-checkpoint, E2F, and MYC targets, indicating active regulation of mitosis and transcription. “Analyzer_Normal” also shows strong enrichment for MYC and E2F targets, further supporting that the normal cell-specific expression includes tightly regulated growth-associated programs.

Mixed intersections—those involving combinations of both cancer and normal datasets—contribute smaller gene groups compared to the large single-source intersections but show a high density of functional enrichment. For instance, the intersection “Analyzer_Cancer.Analyzer_Normal.GTEx_Normal” contains 94 genes and is enriched in multiple hallmark pathways, including E2F targets and G2M checkpoint, reflecting strong involvement in cell cycle regulation. Similar enrichment is observed in the five-way intersection that includes all datasets and contains 39 genes, and the two four-way intersections containing 37 and 10 genes, respectively (Fig. 10).

From the comparison of the enrichment results across cancer-specific, normal-specific, and mixed intersections, distinct patterns emerge. Normal cell-specific gene sets are strongly enriched in core proliferative and transcriptional programs, including E2F, G2/M checkpoint, and MYC targets. These reflect tightly regulated growth-associated functions active in normal tissues. Cancer cell-specific intersections are enriched in pathways such as coagulation and E2F targets, suggesting a shift toward deregulated cell cycle control and tumor-associated processes. Interestingly, mixed intersections involving both cancer and normal datasets also show strong enrichment in proliferation-related pathways, including E2F and MYC targets, and the G2/M checkpoint. The enrichment of these pathways in intersections containing both normal and cancer datasets may suggest that such genes are part of essential regulatory networks that remain active across states or are particularly responsive to changes in POLE2 expression. Their consistent correlation with POLE2 in cancer implies that when POLE2 is dysregulated, these genes may be co-altered as part of broader transcriptional shifts.

To identify genes strongly correlated with POLE2 in a cancer-specific manner and to explore patterns that may distinguish one TCGA cancer type from others, an intersection analysis was performed across all available TCGA cancer types using five expression sources: GEPIA_Cancer, UALCAN_Cancer, Analyzer_Cancer, GTEx_Normal, and Analyzer_Normal. Since Correlation AnalyzeR and GTEx datasets are tissue-based and not cancer-type-specific, their mappings to TCGA cancer types were adapted using the cancer classification used in GEPIA2, with minor adjustments to ensure consistency.

Initially, 23 TCGA cancer types were available across the datasets. However, to ensure meaningful intersection analysis, only cancer types with at least three represented datasets were included. As shown in the Supplementary Fig. S8, the final selection includes 10 cancer types: KICH, KIRC, KIRP, LAML, STAD, SKCM, LUAD, ESCA, DLBC, and GBM.

The genes found in cancer-specific intersections (Supplementary Fig. S8) were subject to enrichment analysis independently for each cancer type to explore their associated biological functions (Fig 11).

**Figure 11.**
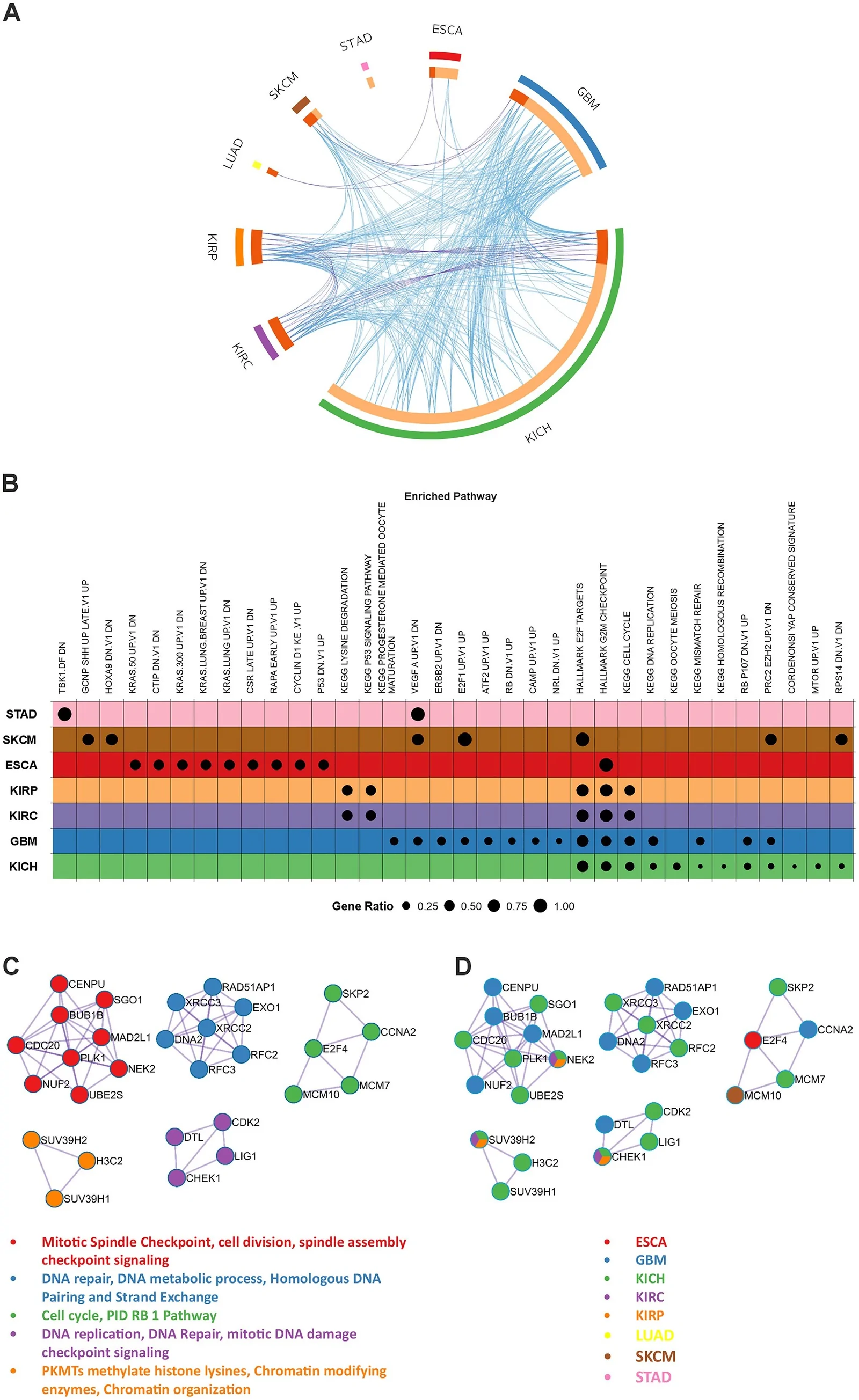
Enrichment analysis of gene groups exclusively correlated with POLE2 in cancer. (**A**) Circos plot retrieved from Metascape showing overlaps among genes that share the same enriched ontology term(s). Purple curves connect identical genes, while blue curves link genes associated with the same ontology term. (**B**) Over-representation analysis of gene groups exclusively correlated with POLE2 in TCGA cancers. Only significant categories (*P*-value < .05) are shown. Gene Ratio refers to the proportion of input genes associated with a particular enriched term. (**C**) PPI network from Metascape for all cancer types merged and colored by MCODE functional clusters. (**D**) PPI network from Metascape for all cancer types merged and colored by cancer types—names of initial gene groups.

The Circos plot (Fig. 11A) illustrates shared GO terms among the POLE2-correlated gene sets from different cancer types. Many shared genes are observed among the three kidney cancers—KIRC, KIRP, and KICH—which reflects their common tissue origin, as they were all mapped from kidney-specific expression data. GBM and KICH appear to share the highest number of POLE2-correlated genes with other cancer types.

The dot plot (Fig. 11B) summarizes the ORA results for gene sets exclusively correlated with POLE2. While enrichment analysis was performed for 11 cancer types, only 7 of them are represented in the plot, as they showed significant enrichment in at least one pathway. The enrichment analysis reveals distinct biological processes across several cancer types. Individual enriched genes from overexpression analysis for each cancer type are shown on the sunburst plot (Supplementary Fig. S9).

Gene sets related to DNA replication and repair include DNA replication and mismatch repair for GBM and KICH, as well as homologous recombination processes for KICH. However, each cancer type has different sets of enriched genes in the same term. For example, DNA replication in GBM contains PRIM1, DNA2, and RFC3, while KICH is enriched with LIG1, RNASEH2A, MCM7, and RFC2. As for mismatch repair, in GBM it includes RFC3 and EXO1, while in KICH it includes RFC2 and LIG1. Homologous recombination in KICH includes XRCC2 and XRCC3.

Enrichment of the gene set linked to epigenetic regulation (“PRC2 EZH2 UP.V1 DN”) [91] was observed in GBM, KICH, and SKCM. This set consists of the 200 most downregulated genes following EZH2 knockdown. In GBM, the overlapping genes include PRIM1, DLGAP5, CENPU, and DNA2. In KICH, the enriched genes are OIP5, SKP2, XRCC3, APLP1, and PSRC1, while in SKCM, the overlapping genes are MCM10 and DLGAP5.

PRC2 (Polycomb Repressive Complex 2) is a key epigenetic regulator involved in transcriptional repression through histone modification. EZH2 is the catalytic subunit of PRC2, responsible for histone methylation at lysine 27 (K27) on histone H3 and lysine 26 (K26) on histone H1 [91]. In GBM, KICH, and SKCM, gene sets that are downregulated upon EZH2 knockdown were found to be enriched. These genes are highly correlated with POLE2 expression and are positively regulated by EZH2, suggesting a potential coordination between POLE2-associated transcriptional programs and EZH2-mediated chromatin regulation in these cancers.

Another gene set linked to epigenetic regulation and associated with ribosomal function and stress response (“RPS14 DN.V1 DN”) [92] was enriched in KICH and SKCM. This set includes genes downregulated upon RPS14 knockdown. In KICH, the overlapping genes are SUV39H2, ESPL1, CDC20, and PLK1. In SKCM, the enriched genes include MCM10 and DLGAP5. SUV39H2 was also identified as a correlated gene of the lysine degradation pathway enriched in both KIRC and KIRP. SUV39H2 encodes a histone methyltransferase involved in lysine modification and heterochromatin formation, linking the pathway to epigenetic regulation.

RPS14 (Ribosomal Protein S14) is a component of the 40S small ribosomal subunit and plays essential roles in ribosome biogenesis, protein synthesis, and broader cellular regulatory pathways. Its loss disrupts the formation of the small ribosomal subunit, causing nucleolar stress and activating the p53 pathway, which can lead to cell cycle arrest and apoptosis [93]. The enrichment of downregulated genes upon RPS14 knockdown was detected in KICH and SKCM. This could indicate that in those cancers, the POLE2-correlated transcriptional program includes genes that are normally suppressed under ribosomal stress, potentially reflecting a state of sustained proliferation despite p53 signaling or ribosomal disruption. In this context, POLE2 may be associated with transcriptional programs that help override growth-inhibitory responses, maintaining expression of mitotic regulators such as CDC20, PLK1, or MCM10 even under conditions that would typically lead to their downregulation.

The most abundant gene sets were associated with cell cycle regulation and mitotic control and were enriched across different cancer types. GBM, KICH, KIRC, and KIRP show enrichment for E2F targets, G2M checkpoint, and cell cycle processes. SKCM also shows enrichment in E2F targets. In GBM and KICH, the “RB_P107_ DN.V1_UP” signature was also enriched. The p53 signaling pathway gene set was enriched in KIRC and KIRP, each with CHEK1 as a correlated gene.

Given that genes that exclusively correlate with POLE2 in cancer overlap with those upregulated after RB knockout, it can mean that those genes are normally inhibited by RB presence. These data confirm that the expression of POLE2-correlated genes is regulated by the E2F transcription factor.

ESCA shows enrichment for the G2M checkpoint gene set as well as the “CYCLIN_D1_KE.V1_UP” gene set, composed of genes upregulated in cells overexpressing a mutant form of Cyclin D1, and the “P53_DN.V1_UP” gene set, which includes genes upregulated in the context of TP53 loss or mutation.

To summarize, this analysis highlights the involvement of POLE2-correlated genes in key cellular processes. Using cancer signature gene sets proved especially valuable, as it allowed observation of how these genes behave across different cancer types and experimental conditions. While many enrichment terms appeared significant in multiple cancers, the specific genes contributing to these terms varied considerably. This suggests that although POLE2 is associated with similar biological processes, the exact gene networks involved are cancer-specific.

As part of functional enrichment in Metascape, a PPI network was constructed, which revealed five distinct clusters (Fig. 11C and D), each corresponding to core biological processes implicated in cancer.

The first cluster, enriched in mitotic spindle checkpoint and cell division genes, formed a tightly connected module. These genes are predominantly associated with kidney tumors, mostly KICH (CDC20, SGO1, PLK1, UBE2S, NEK2), as well as GBM (CENPU, BUB1B, MAD2L1, NUF2). CDC20 is functionally connected to all other genes in this module, suggesting that it might be the main functional driver for deregulation in cell division.

The second cluster involved genes regulating DNA repair, DNA metabolic processes, and homologous recombination. These were strongly linked to GBM (RAD51AP1, EXO1, DNA2, RFC3) and KICH (XRCC3, XRCC2, RFC2).

The third cluster, centered around cell cycle control and E2F regulation, showed overlap across cancers including GBM (CCNA2), KICH (SKP2, MCM7), ESCA (E2F4), and SKCM (MCM10).

The fourth module was enriched in DNA replication, DNA repair, and checkpoint signaling, particularly active in KICH (CDK2, LIG1, CHEK1), KIRC, KIRP (CHEK1), and GBM (DRL), reflecting their reliance on replicative stress responses. These genes partially bridge the DNA repair and cell cycle modules, reinforcing their role as key integrators of cell fate decisions.

Lastly, a distinct chromatin regulation cluster (e.g., SUV39H1, SUV39H2, H3C2) was specifically enriched in KICH, KIRP, and GBM. The lack of direct connections to other clusters may indicate that chromatin state changes act as independent modulators of oncogenesis in these contexts.

A PPI network was constructed using genes exclusively and highly correlated with POLE2 across multiple cancers to uncover functionally coherent clusters involved in key processes like cell division, DNA repair, and chromatin regulation. This helped reveal cancer-specific patterns of gene co-regulation, offering insights into potential mechanisms of POLE2 involvement in tumor biology. Further investigation is needed to determine whether changes in POLE2 could influence these processes by altering the expression of the identified genes. This could help clarify POLE2’s functional role in tumor progression and its potential as a therapeutic target.

## Conclusions

This study integrates multiple data sources to provide more robust insights into how the expression of POLE2, the non-catalytic subunit of DNA polymerase ε, relates to survival and tumor progression across individual cancer types (Fig. 12). We conclude that POLE2 is consistently upregulated across multiple cancer types. Moreover, it stands out as the most overexpressed subunit within the group of replicative polymerases. Its expression levels, rather than CNV, emerge as a more reliable prognostic marker. Stage-dependent expression of POLE2 further suggests a role in tumor progression in certain cancers, such as ACC and KIRC, while others show less pronounced or no consistent trend. Additionally, analysis of mutations identified in POLE2 in cancer samples suggests that they cluster in a specific region. Previous studies in the yeast model showed that mutations in the POLE2 homolog, *DPB2*, affect the Dpb2 protein interactions with other replisome subunits, especially the catalytic subunit of DNA polymerase ε [12, 13, 17]. This highlights the need to further investigate the significance of identified mutations. This study also demonstrates that POLQ stands out as another polymerase with consistent upregulation, linked to replication stress tolerance. In contrast, several translesion polymerases are broadly downregulated.

**Figure 12.**
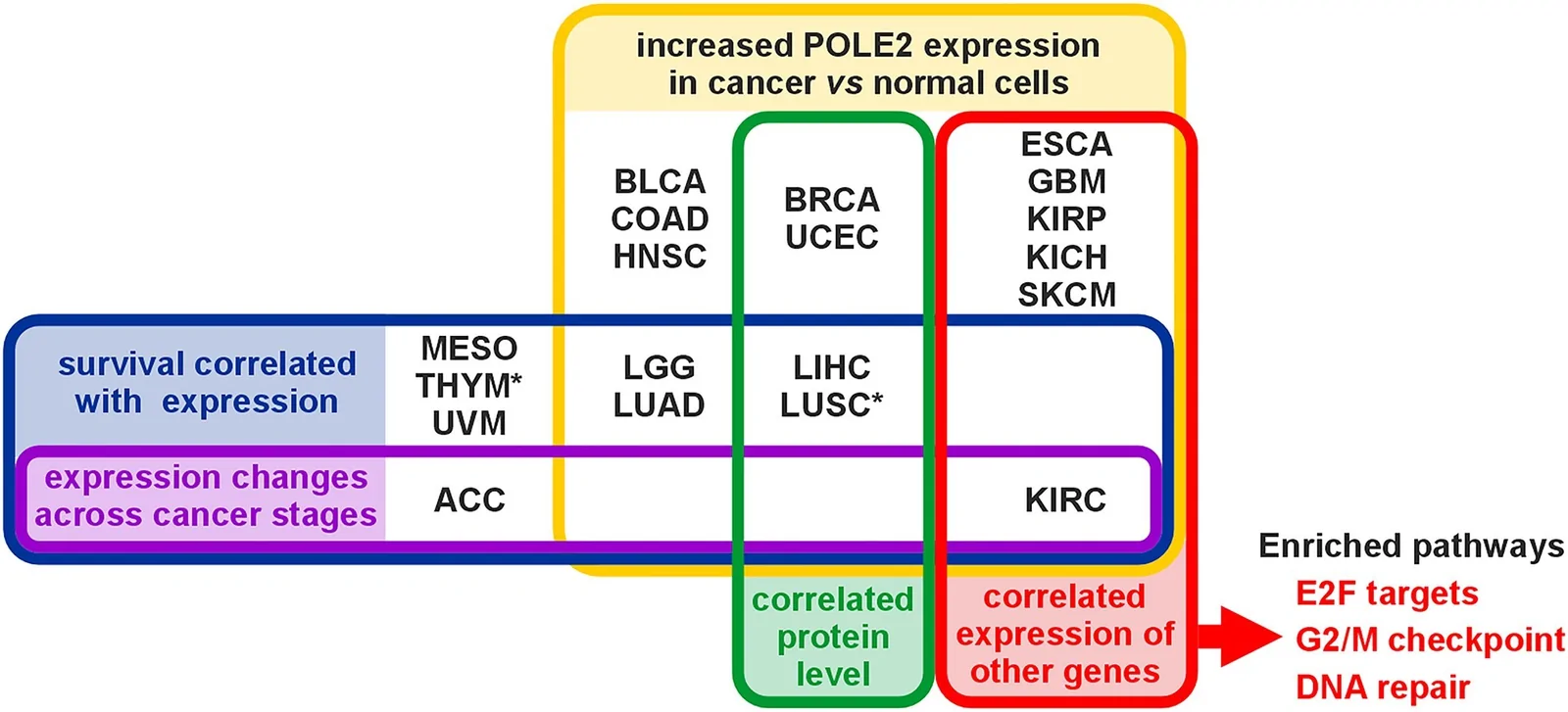
Summary of the most pronounced associations of POLE2 expression with cancer cell physiology. POLE2 expression is upregulated across multiple cancer types (yellow). Moreover, depending on the cancer type, this correlates with a confirmed increase in protein level (green), a specific cancer stage (purple), or increased expression of other genes (red), mainly E2F targets and those involved in the G2/M checkpoint or DNA repair. In many cases, increased POLE2 expression correlates (blue) with poor survival prognosis, but in two cases [*] the effect is inverted.

Elevated POLE2 transcript levels are associated with decreased overall survival (OS) in a cancer-type-specific manner, with particularly strong prognostic significance observed in ACC, LGG, LUAD, MESO, and potentially UVM, KIRC, and LIHC. In contrast, a protective effect was seen in THYM and LUSC, underscoring the context-dependent nature of its role. Despite CNVs influencing POLE2 expression, additional regulatory mechanisms, including promoter methylation, isoform usage, and epigenetic regulators like EZH2, contribute to its complex regulation. The abundance of non-coding isoforms, coupled with the distinct ways coding and non-coding transcripts influence patient survival, highlights the need for isoform-specific studies. Interestingly, POLE2 is tightly co-expressed with genes involved in DNA replication and repair, cell cycle progression, and chromatin remodeling. Finally, PPI network and enrichment analyses indicate that POLE2 is embedded in cancer-specific transcriptional programs that may help override normal growth-inhibitory signals, potentially mediated by E2F, EZH2, and RB pathways. These findings collectively position POLE2 as a functionally significant and clinically relevant marker, with a role that extends beyond DNA replication, and warrant further investigation into its biological function and therapeutic potential in oncology.

## Supplementary Material

lqag118_Supplemental_Files

## Data Availability

The data underlying this article are available in BioStudies at https://www.ebi.ac.uk/biostudies/studies/S-BSST3358, DOI: 10.6019/S-BSST3358.
